# Effects of short-bout accumulated physical activity and sedentary-break interventions on continuous glucose monitoring metrics in adults with type 2 diabetes: A systematic review and meta-analysis

**DOI:** 10.3389/fendo.2026.1901259

**Published:** 2026-09-11

**Authors:** Sicheng Cao, Haozhe Wang, Yongliang Zhu, Changkun Shan, Wenjia Chen

**Affiliations:** 1 School of Physical Education, China University of Mining and Technology, Xuzhou, China; 2 School of Physical Education, Shandong Sport University, Jinan, China; 3 School of Public Health, Jinan Vocational College of Nursing, Jinan, China

**Keywords:** continuous glucose monitoring, exercise snacks, glycemic variability, meta-analysis, sedentary breaks, short-bout accumulated physical activity, systematic review, type 2 diabetes

## Abstract

**Objective:**

Glycated hemoglobin does not fully capture postprandial hyperglycemia or intraday glycemic fluctuation, whereas continuous glucose monitoring (CGM) characterizes mean glucose, hyperglycemic exposure, and glycemic variability. Short-bout accumulated physical activity and sedentary-break interventions are low-burden strategies that can be integrated into daily routines, but their overall effects on CGM-derived outcomes in adults with type 2 diabetes remain uncertain. We systematically reviewed and quantitatively synthesized these effects.

**Methods:**

We searched 11 bibliographic and registry sources from inception to 29 July 2026, after an initial eight-database search through 4 June 2026. Randomized and controlled crossover trials were eligible. We retained the original broad eligibility criteria and examined a narrower *post-hoc* intervention definition in sensitivity analysis. Paired mean differences and standard errors were extracted or reconstructed. Random-effects meta-analysis used restricted maximum likelihood with Hartung–Knapp–Sidik–Jonkman inference. When paired covariance was unavailable, *r* = 0.50 was used, with *r* = 0.25 and *r* = 0.75 sensitivity analyses. Risk of bias was assessed for specific results with crossover RoB 2 and certainty was assessed with GRADE.

**Results:**

Thirteen studies were included; 10 studies (175 participants) contributed to the overall mean-glucose synthesis. Short-bout activity reduced mean glucose (MD = −0.71 mmol/L, 95% CI −1.28 to −0.13; *p* = 0.021), with considerable heterogeneity (*I*² = 91%; tau² = 0.55) and a prediction interval that included the null value (no effect) (−2.48 to 1.06). Intervention category did not explain heterogeneity (*p* = 0.628). All leave-one-out estimates remained significant; omitting Dempsey (2017) reduced *I*² to 5.8% and yielded MD = −0.33 mmol/L (95% CI −0.50 to −0.16). Mean amplitude of glycemic excursions (MAGE) favored activity, whereas glucose standard deviation (SD), coefficient of variation (CV), and time in range (TIR) were inconclusive. Hyperglycemia time was pooled only for compatible thresholds and windows. Certainty ranged from moderate to very low and was low for mean glucose.

**Conclusion:**

Short-bout accumulated physical activity may reduce CGM-derived mean glucose in adults with type 2 diabetes. Although effects remained favorable across correlation, model, arm-selection, and leave-one-out analyses, considerable heterogeneity and a prediction interval that included the null value (no effect) limit confidence in the effect expected in a new setting. Evidence for other glycemic outcomes and safety remains uncertain.

**Systematic Review Registration:**

www.crd.york.ac.uk/prospero, identifier CRD420261415265.

## 1 Introduction

Diabetes is a major global chronic metabolic disease. The 11th edition of the International Diabetes Federation Diabetes Atlas estimated that approximately 589 million adults aged 20–79 years were living with diabetes in 2024, and this number is projected to increase to approximately 853 million by 2050 (1). Long-term hyperglycemic exposure is continuously associated with the risk of microvascular and macrovascular complications in type 2 diabetes (2). Current consensus recommendations emphasize individualized management that integrates lifestyle modification, pharmacotherapy, weight management, and cardiorenal risk protection within a unified care framework (3). Glycated hemoglobin (HbA1c) remains a central marker of long-term glycemic control, but similar HbA1c levels can correspond to markedly different intraday glucose profiles. Reliance on HbA1c alone may therefore obscure postprandial hyperglycemia, hypoglycemia, and glycemic variability (4), while acute glucose fluctuations may also be associated with increased oxidative stress (5).

Continuous glucose monitoring (CGM) and intermittently scanned or flash glucose monitoring (FGM) provide continuous interstitial glucose data and allow evaluation of mean glucose, time in range (TIR), time above range, mean amplitude of glycemic excursions (MAGE), standard deviation (SD), coefficient of variation (CV), and postprandial incremental area under the curve (iAUC). These indices capture different aspects of glycemic exposure and variability, but they also differ in calculation method, interpretive boundary, and dependence on the monitoring window (6). International consensus statements have promoted standardized definitions and reporting of CGM metrics and have highlighted their value in complementing HbA1c and interpreting dynamic glycemic exposure (7, 8). CGM is therefore well suited to evaluating lifestyle interventions that are brief in duration and may exert their main effects during postprandial or other specific intraday windows.

Regular physical activity is recommended for type 2 diabetes management because it can improve glycemic control, reduce cardiometabolic risk, and decrease sedentary exposure (9–11). In practice, however, time constraints, health status, motivation, and environmental barriers often limit adherence to structured exercise programs (12). Sedentary behavior is commonly defined as waking behavior performed while sitting, reclining, or lying with energy expenditure of no more than 1.5 metabolic equivalents (METs) (13), a definition that distinguishes reducing sedentary behavior from simply increasing moderate-to-vigorous exercise. Short-bout accumulated physical activity and sedentary-break interventions include exercise snacks, brief postprandial walking, frequent activity breaks, simple resistance activities, and low-volume interval exercise. These strategies share three features: short bout duration, distributed accumulation, and potential integration into daily routines. Skeletal muscle contraction promotes glucose uptake through insulin-independent pathways, and contraction-related GLUT4 translocation and signaling provide a physiological rationale for short bouts of activity to improve glycemia (14, 15). Trials have also suggested that pre-meal exercise snacks (16) and interrupting sitting with brief walking or simple resistance activities (17) can improve selected postprandial or intraday glycemic metrics.

Broader epidemiological evidence links greater sedentary time with higher risks of diabetes, cardiovascular events, and mortality, with the association with diabetes risk being particularly consistent (18). In acute experimental studies, interrupting sitting with short bouts of walking reduced postprandial glucose and insulin responses in adults with overweight or obesity (19). Among people with newly diagnosed type 2 diabetes, objectively measured sedentary time and breaks in sedentary time were also associated with selected metabolic variables (20). Previous systematic reviews have shown that breaking up sitting can reduce postprandial glucose and insulin responses (21), and exercising more broadly may improve 24-h CGM glucose profiles in people with type 2 diabetes (22, 23). However, these reviews generally included broader populations, intervention types, or study designs and therefore do not specifically answer how short-bout accumulated physical activity and sedentary-break interventions affect multidimensional CGM outcomes in adults with type 2 diabetes. This review therefore focused on adults with type 2 diabetes, short-bout accumulated or sedentary-break interventions, and CGM/FGM outcomes, and systematically evaluated effects on overall mean glucose, glycemic variability, postprandial glycemic responses, and safety.

## 2 Materials and methods

This systematic review and meta-analysis was designed, conducted, and reported in accordance with the Preferred Reporting Items for Systematic Reviews and Meta-Analyses (PRISMA 2020) statement (24), with methods informed by the Cochrane Handbook for Systematic Reviews of Interventions (25). The protocol was prospectively registered with the International Prospective Register of Systematic Reviews (PROSPERO; registration number CRD420261415265; registration URL: https://www.crd.york.ac.uk/PROSPERO/view/CRD420261415265) before formal data extraction. The protocol specified the review question, broad eligibility criteria, outcomes, and planned synthesis. Methodological amendments and additional analyses introduced during the review are identified below and reported with their rationale.

### 2.1 Search strategy

The original search covered eight electronic databases through 4 June 2026 and combined population, intervention, outcome, and study-design concepts. Before finalizing the review, we broadened the strategy to improve sensitivity by removing the outcome concept and expanding coverage to PubMed/MEDLINE, Embase, Scopus, Web of Science Core Collection, CENTRAL, CINAHL Ultimate, APA PsycInfo, SPORTDiscus with Full Text, CNKI, ClinicalTrials.gov, and WHO ICTRP. All revised searches were rerun from database inception on 29 July 2026 using population plus intervention concepts and, where appropriate, a study-design concept. Controlled vocabulary and free-text terms were adapted to each interface. Complete original and revised strategies, interfaces, execution dates, limits, and source counts are provided in the supplementary search material. Reference lists of included reports and relevant reviews were also checked.

### 2.2 Eligibility criteria

Eligibility criteria followed the PICOS framework. Participants were adults with type 2 diabetes. Eligible interventions comprised physical activity delivered in short bouts, accumulated across the day, or used to interrupt prolonged sitting. Comparators included uninterrupted sitting, no exercise, usual activity, or an eligible control condition within the same crossover trial. Eligible studies reported at least one CGM- or FGM-derived outcome and used a randomized or controlled crossover design.

Study selection was based on the original broad qualitative intervention criteria. These criteria were retained for the systematic review. To assess whether the pooled estimates depended on intervention duration and frequency, we conducted a *post-hoc* sensitivity analysis using a narrower operational intervention definition, hereafter termed the *post-hoc* core definition. Exercise snacks and active breaks interrupting sedentary time required active bouts of ≤10 min, at least two bouts per day, and ≤60 min of total active time per day. Accumulated postprandial walking required bouts of ≤20 min, at least two bouts per day, and ≤60 min of total active time per day. Low-volume interval exercise required work intervals of ≤1 min within a complete session of ≤10 min. Standing alone did not satisfy the core intervention definition. This narrower definition did not alter eligibility for the systematic review.

We excluded studies of type 1 diabetes, gestational diabetes, prediabetes, or pediatric diabetes unless data for adults with type 2 diabetes were separately available. We also excluded interventions outside the broad activity concept, conventional longer-duration exercise training, studies without CGM/FGM outcomes, uncontrolled or observational studies, conference abstracts, reviews, case reports, and animal studies. Otherwise eligible studies without a recoverable paired effect were retained in the systematic review but omitted from the relevant quantitative synthesis. Multiple reports from the same trial could inform different outcomes, but no study contributed the same outcome more than once.

### 2.3 Data collection

Two reviewers independently screened records, assessed full texts, and extracted data, with disagreements resolved by discussion or third-reviewer adjudication. Extracted items included age, sex, diabetes duration, body mass index (BMI), HbA1c, medication, randomization and period sequence, washout, intervention bout duration and frequency, total active and standing time, adherence, CGM/FGM device and calibration, sensor completeness, outcome threshold and window, adverse events, and all information required to reconstruct a paired effect.

For crossover trials, the preferred effect was the adjusted paired intervention-control contrast with its SE or CI. When unavailable, paired SEs were derived from a CI, test statistic, or exact *p*-value, followed by a study- or outcome-specific within-person correlation when reported. Study authors were contacted by email to request missing paired-effect or covariance information. When no study-specific coefficient was available, *r* = 0.50 was used, with *r* = 0.25 and *r* = 0.75 sensitivity analyses. Glucose reported in mg/dL was converted to mmol/L by dividing by 18. All calculations used unrounded values. Time in hyperglycemia was pooled only when the glucose threshold, observation window, and denominator were compatible. Results based on 22-h monitoring were analyzed separately and were not treated as 24-h measurements.

To avoid double-counting a shared control group, each multi-arm study contributed one intervention-control comparison per outcome to the main analysis. For each outcome, the eligible arm that most closely matched the review’s intervention concept was selected; ties were resolved by order of appearance in the Methods section. This hierarchy was applied independently of effect magnitude. Every other eligible arm replaced the selected arm one at a time in sensitivity analyses. Study-level selections, alternatives, and quantitative intervention boundaries are reported in the **Supplementary Material**.

### 2.4 Risk of bias and certainty of evidence

Risk of bias was assessed for a specific result and time point with the Cochrane RoB 2 tool for crossover trials. In addition to the standard domains, period effects, carryover, washout, and the analysis of paired observations were considered explicitly. Lack of participant or provider blinding was not treated as sufficient by itself to downgrade the deviations-from-intended-interventions domain; judgments instead considered whether awareness caused deviations that could affect the result. CGM/FGM outcomes were objectively recorded. GRADE judgments considered explicit clinical-importance thresholds and optimal information size (OIS), and statistical significance or whether a CI crossed zero was not used as a stand-alone imprecision rule (26, 27).

### 2.5 Data analysis

The primary synthesis used paired mean differences and SEs in a generic inverse-variance random-effects model. Between-study variance was estimated by REML, and uncertainty was quantified with HKSJ inference. We reported MD, 95% CI, *p*-value, Cochran’s *Q*, *I*², tau², and a 95% prediction interval when estimable. Negative estimates favor the activity intervention, except for TIR, where positive estimates favor intervention. Analyses were performed in R 4.5.3 with metafor 5.0-1 and independently reproduced with meta 8.5-0. A paired-effect DL/normal generic inverse-variance analysis was calculated in R as a conventional sensitivity analysis and is compatible with the RevMan generic inverse-variance framework (28, 29).

Sensitivity analyses comprised DL/normal and REML/normal inference, assumed within-person correlations of 0.25, 0.50, and 0.75, leave-one-out analyses, restriction to the *post-hoc* core definition, and one-at-a-time replacement of each selected arm in multi-arm trials. The formal subgroup analysis classified interventions by clinical delivery pattern: exercise snacks/low-volume intervals, sedentary/activity breaks, or accumulated postprandial walking. To address real-world applicability, a separate *post-hoc* exploratory analysis compared controlled laboratory studies with free-living or mixed-setting studies. Subgroup differences were evaluated with small-sample-adjusted meta-regression; conclusions were based on the interaction test rather than significance within individual subgroups. Because 10 studies contributed to the primary outcome, a contour-enhanced funnel plot and mixed-effects Egger-type regression were undertaken as exploratory small-study-effect analyses. Postprandial iAUC results with incompatible units or windows were not forced into a pooled estimate (30).

## 3 Results

### 3.1 Study selection

**Figure 1:**
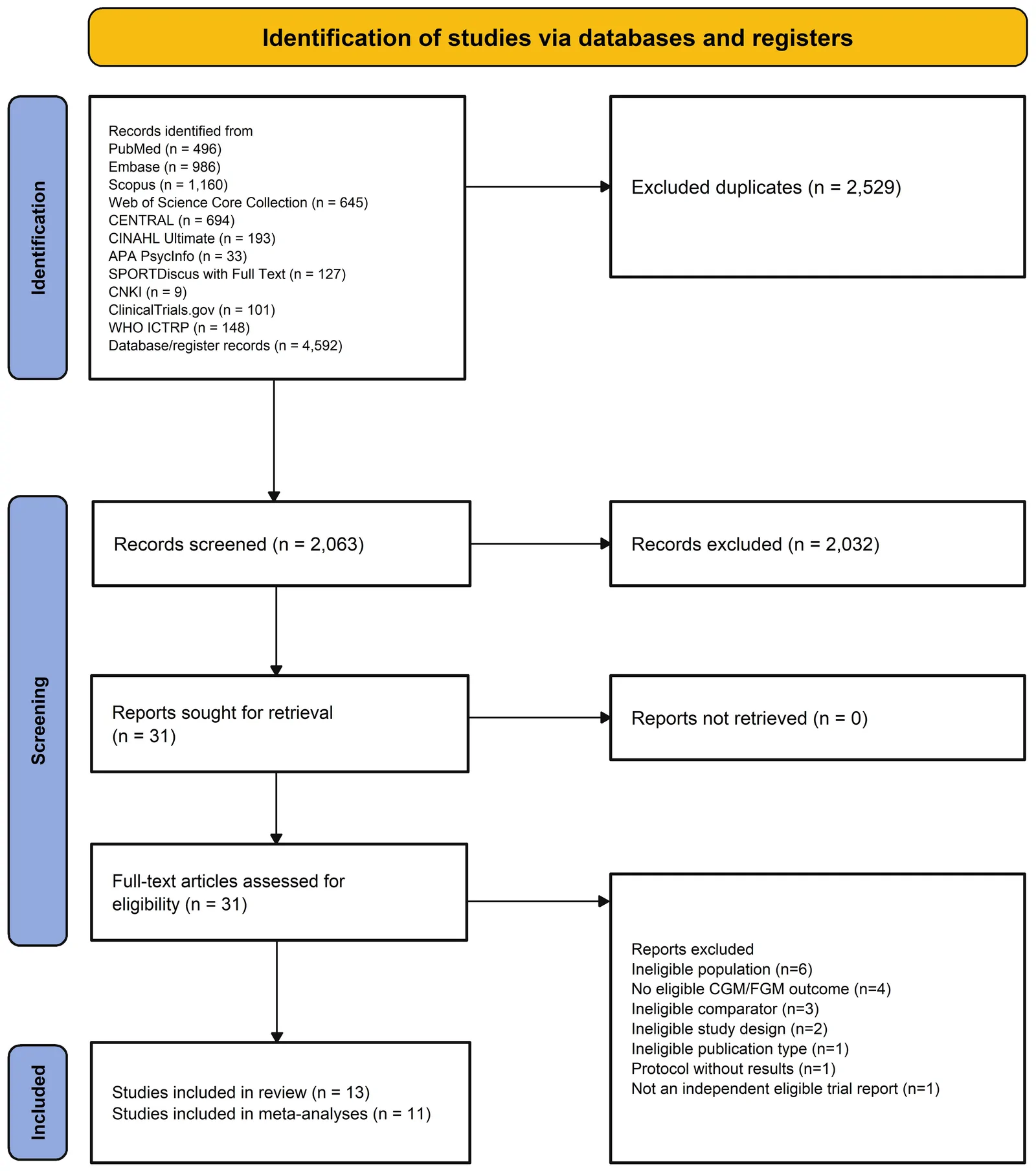
PRISMA 2020 flow diagram for the updated search. The 11 bibliographic and registry sources yielded 4,592 records; 2,529 duplicates were removed, 2,063 unique records were screened, 31 full-text reports were assessed, 18 reports were excluded, and 13 reports representing 13 studies were included. Ten studies contributed to the primary mean-glucose meta-analysis.

The updated searches identified 4,592 records: PubMed (*n* = 496), Embase (*n* = 986), Scopus (*n* = 1,160), Web of Science Core Collection (*n* = 645), CENTRAL (*n* = 694), CINAHL Ultimate (*n* = 193), APA PsycInfo (*n* = 33), SPORTDiscus with Full Text (*n* = 127), CNKI (*n* = 9), ClinicalTrials.gov (*n* = 101), and WHO ICTRP (*n* = 148). After 2,529 duplicates were removed, 2,063 unique records underwent title and abstract screening. Thirty-one reports were assessed in full text. Eighteen reports were excluded for specified eligibility reasons, and 13 reports representing 13 studies were included. Ten studies contributed to the primary mean-glucose meta-analysis (**Figure 1**).

### 3.2 Study characteristics

Thirteen crossover or controlled within-person studies published from 2012 to 2026 enrolled 251 adults with type 2 diabetes; 10 studies (175 participants) contributed to the primary synthesis. Samples ranged from 7 to 41 participants. Several studies enrolled only or predominantly men, whereas Marcotte-Chénard (2024) (43) enrolled only women. Most excluded insulin-treated participants or people unable to undertake exercise. Mean ages were approximately 52–70 years, and populations generally had stable, reasonably controlled type 2 diabetes. Applicability is therefore greatest to non-insulin-treated adults able to complete short activity bouts and is more limited for insulin users, people with advanced complications, frailty, or exercise contraindications (31–43).

The interventions comprised activity breaks, accumulated postprandial walking, exercise snacks, and low-volume intervals. Seven primary-outcome comparisons met the *post-hoc* core definition. Duvivier (2017) (34) exceeded the 60 min/day active-time boundary; Gillen (2012) (39), Terada (2016) (41), and Marcotte-Chénard (2024) (43) exceeded the complete-session boundary for low-volume intervals. de Hoogh (2025) (42) and Marcotte-Chénard (2024) (43) were retained in the qualitative synthesis but lacked a recoverable paired marginal effect for quantitative pooling; van Dijk (2013) (38) also lacked a reliable paired mean-glucose effect. Monitoring windows ranged from 22 h to 4 days. Main characteristics are shown in **Table 1**, with detailed trial conduct and intervention dose in **Supplementary Tables 1**-**3**.

**Table 1:** Basic characteristics of the included studies.

Study	Country	*n*	Women/men	Age, years	Population/therapy	Main intervention dose	Design, device, and window
Babir 2026 (31)	Canada	31	21/10	58 (11)	Inactive; non-insulin-treated	Four 1-min vigorous snacks/day	Real-world crossover; Libre 2; 48 h
Blankenship 2019 (32)	USA	30	16/14	64 (8.2)	Sedentary; medication continued	12 postmeal breaks/day; 20/40/60 min total	Free-living crossover; iPro2; 24 h
Dempsey 2017 (33)	Australia	24	10/14	62 (6)	Inactive; diet/metformin controlled	3 min walking every 30 min; 36 min	Laboratory crossover; iPro2; 22 h
Duvivier 2017 (34)	Netherlands	19	6/13	63 (9)	Non-insulin-treated	~132 min walking plus ~150 min standing/day	Free-living crossover; iPro2; 24 h
Haxhi 2016 (35)	Italy	9	0/9	58.2 (6.6)	Men; oral therapy	Two 20-min walks around lunch	Crossover; iPro; 24 h
Homer 2021 (36)	Australia	24	11/13	62 (8)	Non-insulin therapy	3 min SRA every 30 min; 36 min	Laboratory crossover; Libre; 22 h
Metcalfe 2018 (37)	UK	11	0/11	52 (6)	Men; no insulin	Two 20-s sprints in 10 min	Crossover; iPro2; 24 h
van Dijk 2013 (38)	Netherlands	20	0/20	64 (1 SE)	Men; oral therapy	Three 15-min postmeal walks	Crossover; GlucoDay S; 24 h
Gillen 2012 (39)	Canada	7	NR	62 (3)	T2DM; mostly medicated	Ten 1-min HIT bouts; ~25-min session	Fixed-order within-person; 24 h CGM
Myette-Côté 2018 (40)	Canada	11	7/4	64 (8)	Non-insulin-treated	Three 15-min postmeal walks/day	Randomized crossover; iPro2; 4 days
Terada 2016 (41)	Canada	10	2/8	60 (6)	T2DM; outcome *n* = 7–10	HIIEfast: fifteen 1-min work intervals; 60-min session	Randomized crossover; iPro2; 24 h
de Hoogh 2025 (42)	Netherlands	41	19/22	62.3 (7.2)	Insulin-naive; lifestyle/metformin	Active day: 5 min hourly, 9 bouts	Randomized repeated periods; Dexcom G6; 4 days
Marcotte-Chénard 2024 (43)	Canada	14	14/0	70 (4)	Older women with T2DM	HIIT10: ten 1-min intervals; ~38-min session	Semi-randomized crossover; Dexcom G6; 24 h

T2DM, type 2 diabetes mellitus; CGM, continuous glucose monitoring; FGM, flash glucose monitoring; AUC, area under the curve; iAUC, incremental area under the curve; SD, standard deviation; CV, coefficient of variation; MAGE, mean amplitude of glycemic excursions; TIR, time in range; TAR, time above range; TBR, time below range; HRR, heart-rate reserve.

### 3.3 Risk of bias

**Figure 2:**
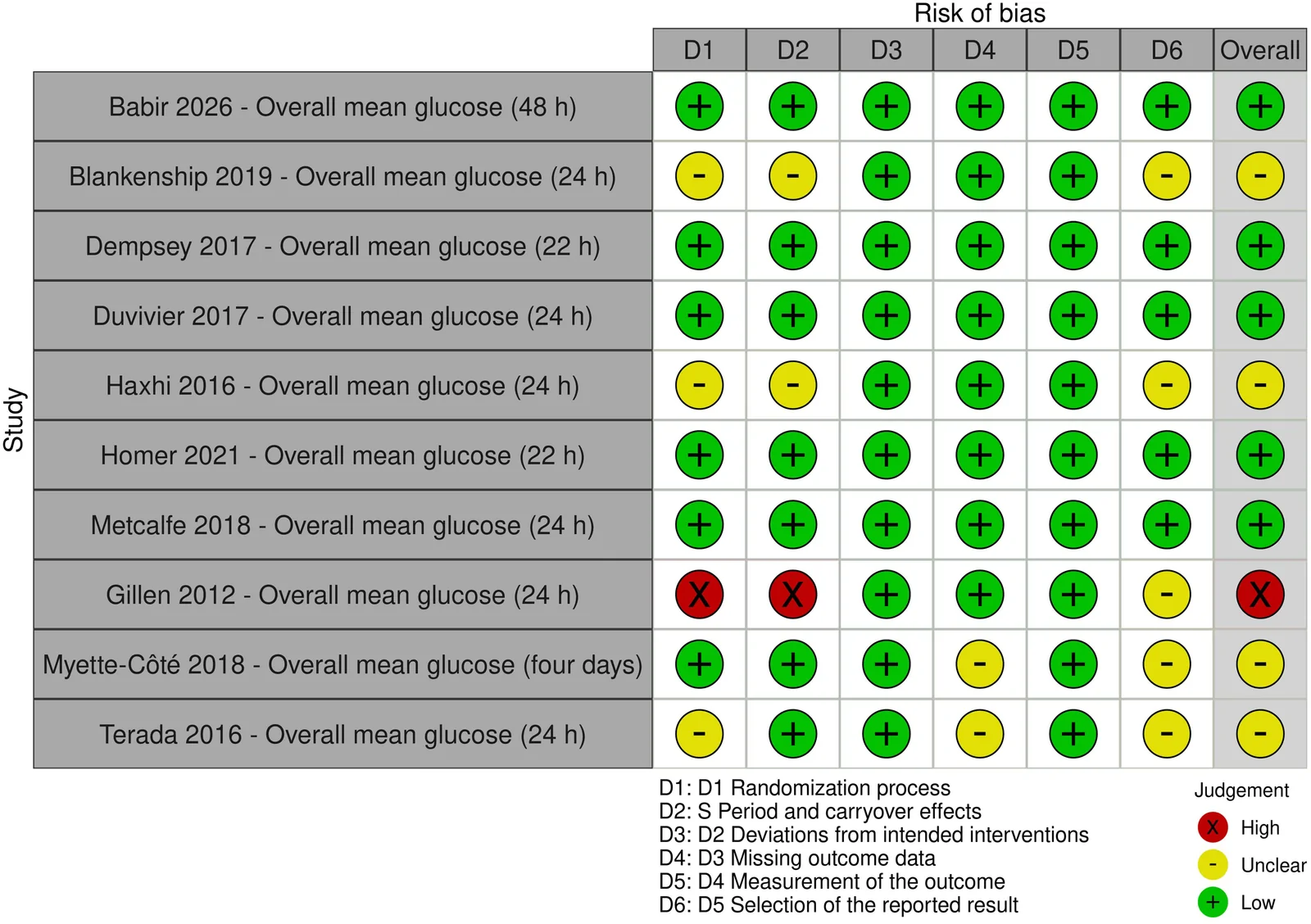
Outcome-specific RoB 2 judgments for the primary mean-glucose result from each of the 10 studies included in the primary meta-analysis. The period/carryover column records crossover-specific concerns. Lack of participant blinding was not used alone to downgrade D2. Green indicates low risk, yellow denotes some concerns, and red indicates high risk; result-level mappings and signaling-question responses are provided in **Supplementary Tables 7**, **10**, **11**.

Risk-of-bias judgments were made for specific results and time points with the crossover RoB 2 framework (**Figure 2**; **Supplementary Tables 7**, **10**, **11**). Among the 10 pooled mean-glucose results, 5 were low risk, 4 had some concerns, and Gillen (2012) (39) was high risk because the control condition was always completed before exercise, confounding treatment with period. Marcotte-Chénard (2024) (43) was also high risk for its non-pooled mean-glucose result because control was fixed first. . . .

Randomization and crossover-period effects: Babir (2026) (31), Dempsey (2017) (33), Duvivier (2017) (34), Homer (2021) (36), Metcalfe (2018) (37), Myette-Côté (2018) (40), and de Hoogh (2025) (42) provided comparatively detailed sequence procedures. Blankenship (2019) (32), Haxhi (2016) (35), Terada (2016) (41), and van Dijk (2013) (38) provided insufficient information on one or more aspects of sequence generation, concealment, or period/carryover effects. Gillen (2012) (39) and Marcotte-Chénard (2024) (43) used fixed-order control conditions and were rated high risk for this domain.

Deviations from intended interventions: all efficacy results were judged low risk in this domain. Participants necessarily knew the assigned activity condition, but the studies did not show systematic intervention-related deviations likely to bias the objective CGM result, and non-blinding alone was not used as a reason for downgrading.

Missing outcome data: most studies had high completion of crossover conditions. In Homer (2021) (36), nine missing pre-breakfast baseline readings affected meal-specific iAUC calculations but not the 22-h mean-glucose result; the primary result was therefore judged low risk in this domain. Myette-Côté (2018) (40) and Terada (2016) (41) had some concerns because of incomplete crossover data or outcome-specific sample sizes. These issues were separated from non-reporting of an extractable paired contrast.

Outcome measurement: CGM/FGM outcomes were recorded automatically with the same device and procedures across conditions within each study. Awareness of condition was unlikely to influence the recorded glucose values, so efficacy results were judged low risk in this domain.

Selection of the reported result: prospective registration or prespecified analyses were available for several recent studies. Blankenship (2019) (32), Haxhi (2016) (35), Gillen (2012) (39), Myette-Côté (2018) (40), and Terada (2016) (41) lacked equally clear result-specific prespecification and were judged as having some concerns. Safety results were generally more weakly prespecified than efficacy results.

Overall concerns therefore arose mainly from incomplete randomization reporting, period/order confounding, missing CGM/FGM observations, or unclear result-specific prespecification, rather than from the unavoidable absence of participant blinding. GRADE for mean glucose was not downgraded for risk of bias because the high-risk Gillen result contributed only 9.1% of the weight, objective CGM limited measurement bias, and omitting Gillen produced a concordant pooled effect.

### 3.4 Meta-analysis results

#### 3.4.1 Primary outcome: overall mean glucose

**Figure 3:**
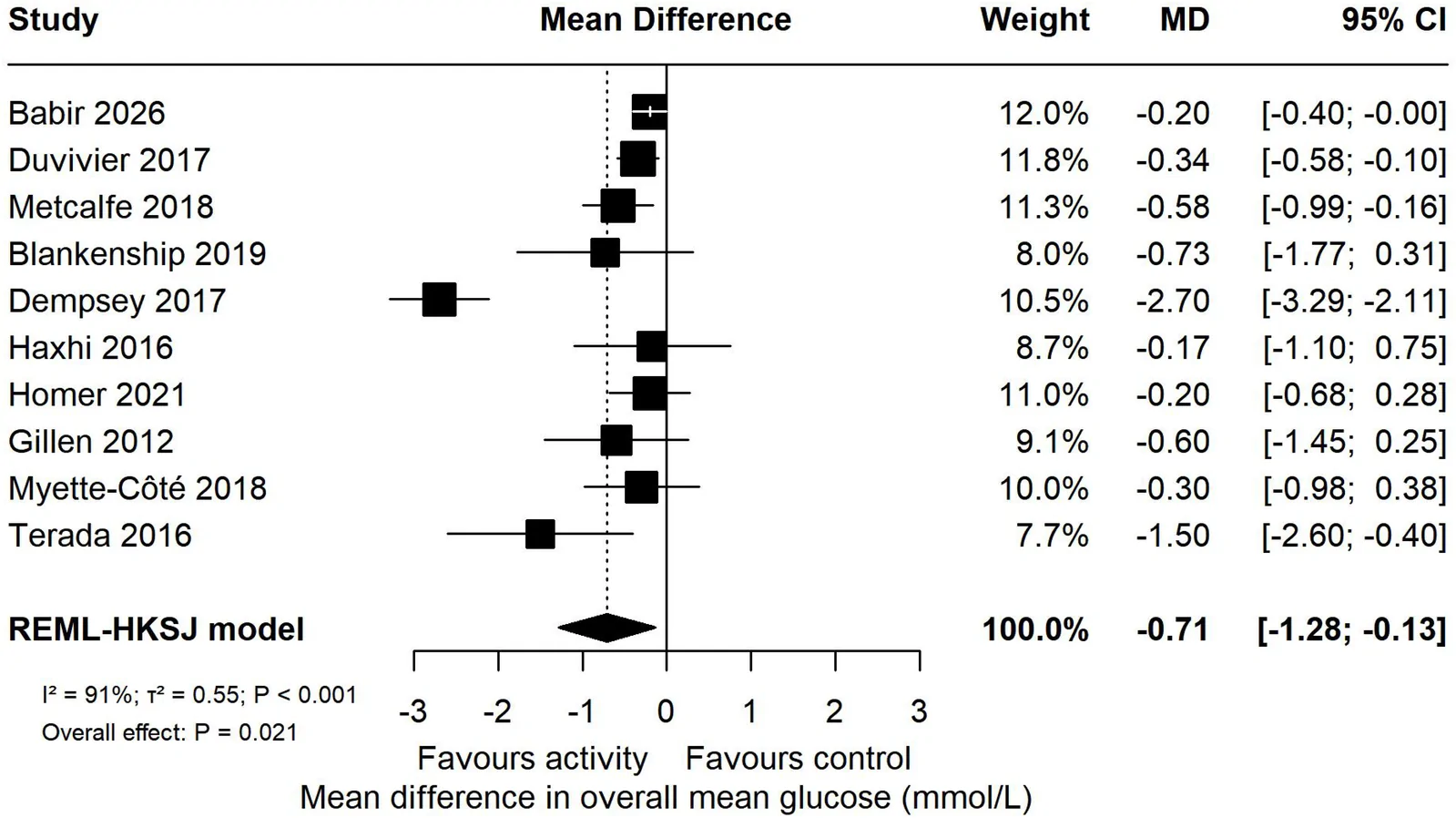
Paired-effect random-effects meta-analysis of overall mean glucose. Study estimates are paired mean differences with 95% CIs. The pooled estimate uses REML heterogeneity estimation and HKSJ inference. Negative values favor activity. The pooled result was MD = −0.71 mmol/L (95% CI −1.28 to −0.13; *p* = 0.021; *I*² = 91.39%; tau² = 0.55).

Ten studies reported an extractable paired effect for overall mean glucose. The authors of five included studies with incomplete paired information were contacted by email. The paired-effect REML-HKSJ model produced MD = −0.71 mmol/L (95% CI −1.28 to −0.13; *p* = 0.021), with *Q* = 68.79 (df = 9; *p* < 0.001), *I*² = 91.39%, tau² = 0.55, and a 95% prediction interval from −2.48 to 1.06 mmol/L (**Figure 3**). The point estimate and most of the confidence interval exceeded the 0.5 mmol/L clinical-importance threshold used for the GRADE assessment, but the prediction interval included no effect and possible effects in either direction. Three additional included studies were not forced into this synthesis because a reliable paired mean-glucose contrast could not be recovered.

**Figure 4:**
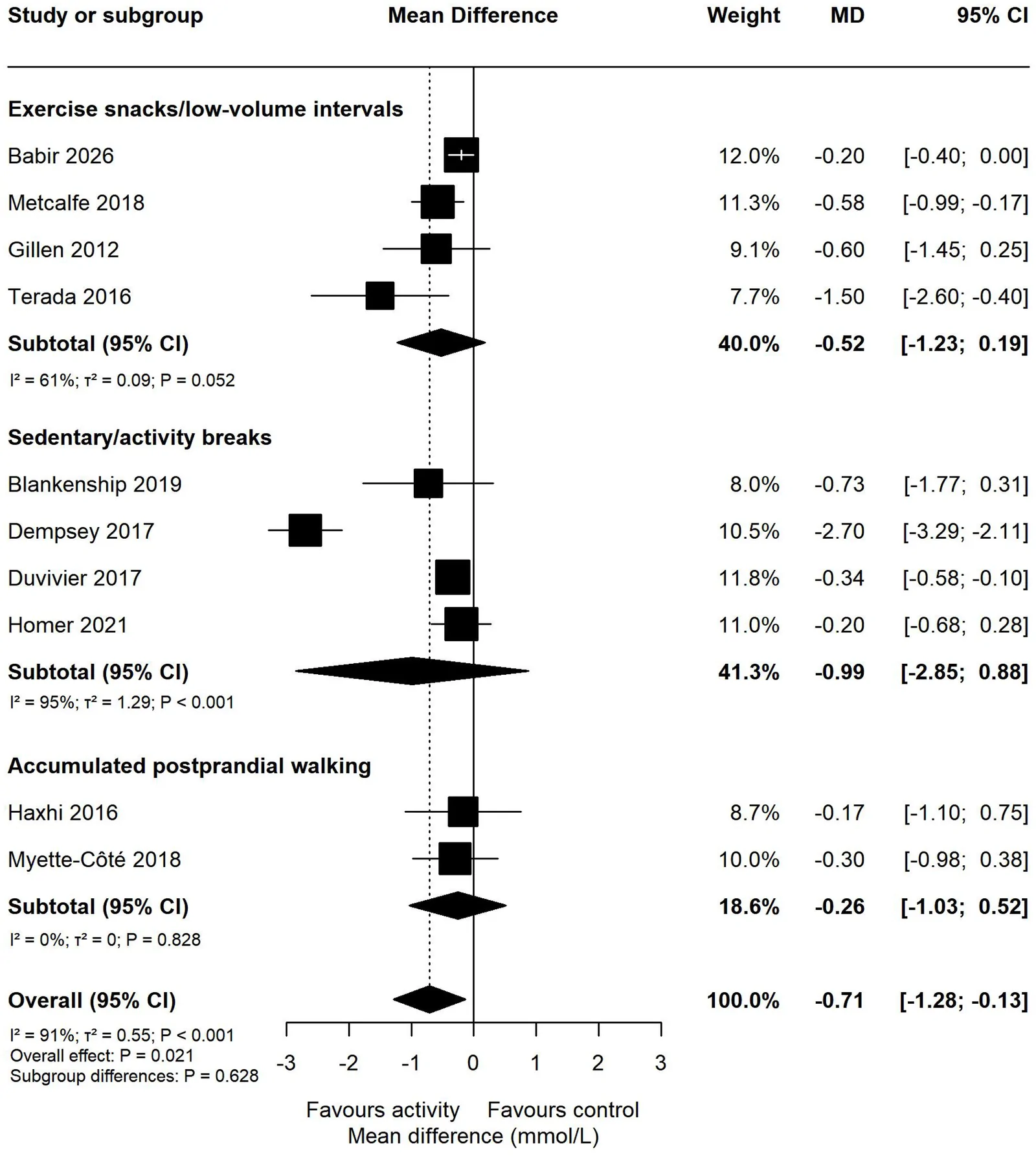
Intervention-category analysis of overall mean glucose using paired effects and REML-HKSJ inference. The moderator test did not show a subgroup difference (*p* = 0.628). Category-specific estimates are exploratory because each subgroup contained only two to four studies.

The intervention-category estimates were −0.99 mmol/L (95% CI −2.85 to 0.88; *k* = 4) for sedentary/activity breaks, −0.52 mmol/L (95% CI −1.23 to 0.19; *k* = 4) for exercise snacks/low-volume intervals, and −0.26 mmol/L (95% CI −1.03 to 0.52; *k* = 2) for accumulated postprandial walking. The small-sample moderator test showed no evidence that category explained heterogeneity (*F*[2,7] = 0.50; *p* = 0.628). These broad and overlapping intervals do not establish comparative superiority (**Figure 4**).

**Figure 5:**
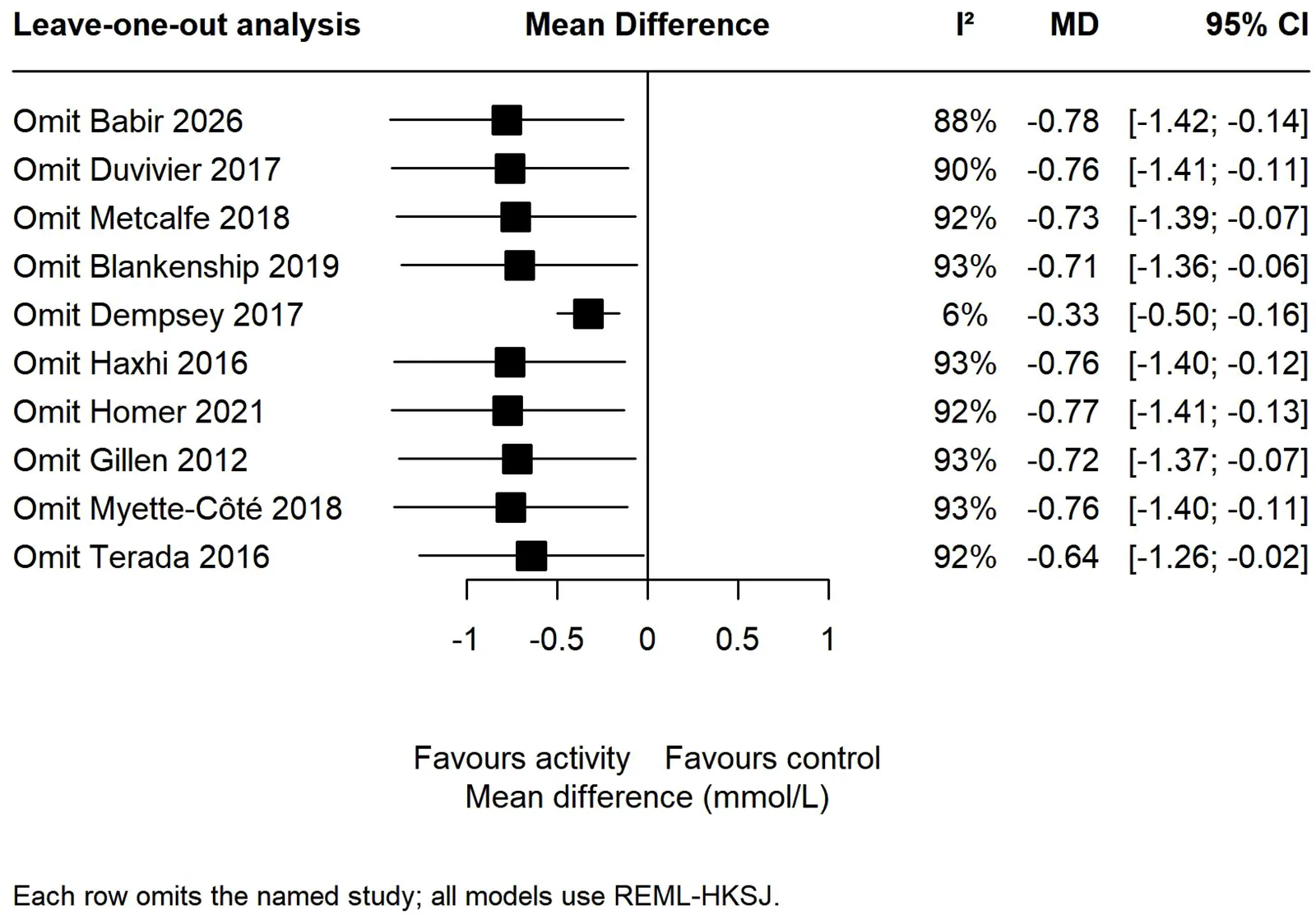
Leave-one-out REML-HKSJ analyses of overall mean glucose. Each row refits the model after omitting the indicated study; negative estimates favor activity.

In the *post-hoc* setting analysis, free-living or mixed-setting studies produced a more consistent estimate (*k* = 5; MD = −0.28 mmol/L, 95% CI −0.43 to −0.13; *I*² = 0%) than controlled laboratory studies (*k* = 5; MD = −1.02 mmol/L, 95% CI −2.38 to 0.33; *I*² = 91.85%). However, the formal interaction was not significant (*F*[1,8] = 1.54; *p* = 0.250), so setting was not established as an effect modifier (**Supplementary Figure 12**; **Figure 5**). All 10 leave-one-out estimates remained statistically significant and favorable (**Figure 5**). Omitting Dempsey (2017) (33) produced the largest change, yielding MD = −0.33 mmol/L (95% CI −0.50 to −0.16; *p* = 0.002) and reducing *I*² from 91.39% to 5.8%. Baujat and influence diagnostics likewise identified Dempsey (2017) (33) as the principal driver (externally studentized residual −7.47; Cook’s distance 2.23). Dempsey was retained because it met eligibility criteria and its exclusion was not prespecified; the diagnostic shows that it explains much of the magnitude and heterogeneity, not that the full-model result is invalid.

The primary effect was stable across assumed correlations (*r* = 0.25: MD = −0.70, 95% CI −1.28 to −0.13; *r* = 0.75: MD = −0.71, 95% CI −1.29 to −0.13), DL/normal inference (MD = −0.70, 95% CI −1.12 to −0.28; *p* = 0.001), and REML/normal inference (MD = −0.71, 95% CI −1.21 to −0.20; *p* = 0.006). Restriction to the *post-hoc* core definition retained a similar point estimate but a wider interval (*k* = 7; MD = −0.70, 95% CI −1.55 to 0.15; *p* = 0.091). All eight one-at-a-time alternative-arm analyses remained significant, with pooled MDs from −0.73 to −0.67 mmol/L. Thus, the main conclusion was not determined by the assumed correlation or selected intervention arm, whereas the narrower core analysis remained imprecise.

#### 3.4.2 Secondary outcomes and safety

MAGE favored activity (six studies; MD = −0.63 mmol/L, 95% CI −1.25 to −0.02; *p* = 0.045; *I*² = 63.54%). Glucose SD (four studies; MD = −0.14 mmol/L, 95% CI −0.44 to 0.16; *p* = 0.225; *I*² = 75.28%), glucose CV (three studies; MD = 0.69 percentage points, 95% CI −3.86 to 5.23; *p* = 0.582; *I*² = 70.59%), and TIR (two studies; MD = 1.88 percentage points, 95% CI −2.07 to 5.82; *p* = 0.104; *I*² = 0%) remained inconclusive. Postprandial iAUC was synthesized narratively because units, meal windows, and calculation methods were not sufficiently comparable. Complete study-level forest plots are provided in **Supplementary Figures 2**-**5**.

Time in hyperglycemia was stratified by threshold and window. For >10 mmol/L over 24 h, five studies yielded MD = −7.26 percentage points (95% CI −11.09 to −3.43; *p* = 0.006; *I*² approximately 0%). Terada (2016) (41) reported minutes and was transparently converted to percentage points by dividing by 14.4; original minute values were retained in the extraction workbook. For >10 mmol/L over 22 h, two studies yielded MD = −20.83 percentage points with an extremely imprecise HKSJ CI (−240.30 to 198.64; *I*² = 97.94%); this was inconclusive. Studies using other thresholds or windows were reported separately and not combined with these strata.

Replacing each selected multi-arm comparison one at a time did not materially alter the primary estimate; all eight REML-HKSJ replacement analyses excluded no effect. Replacement estimates ranged from −0.73 to −0.67 mmol/L. This consistency reduces concern that the main result was created by selecting a favorable arm.

To keep the main manuscript focused and the forest-plot format consistent, complete secondary-outcome plots, model and arm sensitivities, the *post-hoc* setting subgroup, the Baujat plot, influence panels, and contour-enhanced funnel plot are provided in the **Supplementary Material**. The exploratory mixed-effects Egger-type regression found no statistical evidence of small-study effects (*t*[8] = −0.73; *p* = 0.488). Because this analysis was performed at the minimum recommended study count and the primary synthesis was highly heterogeneous, it cannot exclude missing-evidence bias.

Safety reporting was heterogeneous and predominantly descriptive (**Supplementary Table 6**). Dempsey (2017) (33) and Duvivier (2017) (34) reported no hypoglycemic episodes or periods. Babir (2026) (31) observed similar proportions with any time below range (9/31 vs. 10/31). Homer (2021) (36) recorded five asymptomatic CGM-detected episodes across conditions, one of which appeared to be a sensor artifact. Metcalfe (2018) (37) reported one subjective episode during HIIT while CGM remained 5–6 mmol/L. Myette-Côté (2018) (40) documented five non-completers without an exercise-attributed harm; Terada (2016) (41) reported one unrelated injury; de Hoogh (2025) (42) reported one medication-related withdrawal; and Marcotte-Chénard (2024) (43) described brief hypoglycemia in six women without a clear exercise-related excess. Several studies did not systematically report events. Non-reporting was not interpreted as zero events, and evidence is inadequate for a definitive safety conclusion.

Pooled estimates and heterogeneity statistics are summarized in **Table 2**. Full-precision effects, SEs, derivations, and sensitivity analyses are provided in the analysis workbook and **Supplementary Material**.

**Table 2:** Summary of meta-analysis results by outcome.

Outcome	*k* (*n*)	MD (95% CI)	*p*	*Q* (df; *p*)	*I*²	tau²	95% PI	Model
Overall mean glucose, mmol/L	10 (175)	-0.71 (−1.28 to −0.13)	0.021	68.79 (9; <0.001)	91.39%	0.55	−2.48 to 1.06	REML-HKSJ
MAGE, mmol/L	6 (95)	−0.63 (−1.25 to −0.02)	0.045	13.49 (5; 0.019)	63.54%	0.18	−1.88 to 0.62	REML-HKSJ
Glucose SD, mmol/L	4 (75)	−0.14 (−0.44 to 0.16)	0.225	9.83 (3; 0.020)	75.28%	0.03	−0.73 to 0.44	REML-HKSJ
Glucose CV, percentage points	3 (85)	0.69 (−3.86 to 5.23)	0.582	7.7 (2; 0.021)	70.59%	2.65	−7.66 to 9.03	REML-HKSJ
TIR, percentage points	2 (55)	1.88 (−2.07 to 5.82)	0.104	0.05 (1; 0.827)	0%	0	NA	REML-HKSJ
Time >10 mmol/L, 24 h, percentage points	5 (85)	−7.26 (−11.09 to −3.43)	0.006	3.32 (4; 0.506)	0%	0	−11.09 to −3.43	REML-HKSJ
Time >10 mmol/L, 22 h, percentage points	2 (48)	−20.83 (−240.3 to 198.64)	0.441	48.64 (1; <0.001)	97.94%	584.43	NA	REML-HKSJ

Estimates are paired mean differences. REML-HKSJ was used for the primary and outcome-specific random-effects models. PI, prediction interval; NA, not estimable. Negative values favor activity except for TIR. Hyperglycemia strata combine only common thresholds, observation windows, and denominators.

### 3.5 Certainty of evidence

GRADE judgments are summarized in **Table 3**; **Supplementary Tables 8**, **9**. Certainty was not determined from statistical significance alone. For overall mean glucose, risk of bias was not downgraded because objective CGM limited measurement bias, the single high-risk pooled result contributed 9.1% of the weight, and its omission produced a concordant estimate. Inconsistency was downgraded one level because *I*² was 91%, the prediction interval crossed no effect, and Dempsey (2017) (33) strongly influenced heterogeneity. Imprecision was downgraded one level because the CI spanned a smaller and a clinically important benefit and the prediction interval was wide; certainty was low. CV, MAGE, and glucose SD were low certainty; TIR and 24 h >10 mmol/L were moderate certainty; the 22-h stratum and safety were very low certainty. The exploratory Egger-type test did not detect small-study effects, but the minimum study count and high heterogeneity preclude a strong absence-of-bias claim.

**Table 3:** GRADE summary of evidence.

Outcome	Effect (95% CI)	Risk of bias	Inconsistency	Indirectness	Imprecision	Publication bias	Certainty
Overall mean glucose	−0.71 (−1.28 to −0.13)	Not serious	Serious	Not serious	Serious	Not serious; exploratory *p* = 0.488	Low
MAGE	−0.63 (−1.25 to −0.02)	Not serious	Serious	Not serious	Serious	Undetected; *k* < 10	Low
Glucose SD	−0.14 (−0.44 to 0.16)	Not serious	Serious	Not serious	Serious	Undetected; *k* < 10	Low
Glucose CV	0.69 (−3.86 to 5.23)	Not serious	Serious	Not serious	Serious	Undetected; *k* < 10	Low
TIR	1.88 (−2.07 to 5.82)	Not serious	Not serious	Not serious	Serious	Undetected; *k* < 10	Moderate
Time >10 mmol/L, 24 h	−7.26 (−11.09 to −3.43)	Not serious	Not serious	Not serious	Serious	Undetected; *k* < 10	Moderate
Time >10 mmol/L, 22 h	−20.83 (−240.3 to 198.64)	Not serious	Very serious	Not serious	Very serious	Undetected; *k* < 10	Very low
Safety	Not pooled	Serious	NA	Serious	Very serious	Undetected	Very low

CI, confidence interval; CV, coefficient of variation; HKSJ, Hartung–Knapp–Sidik–Jonkman; MAGE, mean amplitude of glycemic excursions; MID, minimally important difference; OIS, optimal information size; TIR, time in range. Mean glucose MID = 0.5 mmol/L; time outcomes MID = 5 percentage points; other continuous outcomes used 0.2 of a representative control-group SD. GRADE judgments incorporated OIS, clinical thresholds, heterogeneity, risk of bias, and search completeness rather than statistical significance alone.

## 4 Discussion

### 4.1 Summary of findings

This review included 13 crossover or controlled within-person studies; 10 contributed paired effects to the primary synthesis, which indicated an approximately 0.71 mmol/L reduction in overall mean glucose. However, substantial residual heterogeneity (*I*² = 91%), a prediction interval that included the null value (no effect), and the influence of Dempsey (2017) (33) show that the pooled estimate is an average across diverse protocols rather than a reliably transportable effect for a particular setting. Sensitivity and leave-one-out analyses remained favorable but do not remove this uncertainty.

MAGE also favored activity, whereas glucose SD, CV, and TIR remained inconclusive. Hyperglycemia results depended on threshold and observation window: the harmonized 24-h >10 mmol/L stratum favored activity, whereas the heterogeneous 22-h stratum was extremely imprecise. Safety data did not show an obvious activity-related signal, but inconsistent ascertainment and non-reporting prevent firm reassurance.

### 4.2 Comparison with previous evidence and potential mechanisms

The direction of the mean-glucose estimate agrees with evidence that physical activity can improve glycemic exposure, but 10 small crossover studies cannot define the effect expected in every setting. The subgroup analyses were exploratory and contained only two to four studies per intervention category. Neither intervention category (*p* = 0.628) nor the *post-hoc* laboratory-versus-free-living comparison (*p* = 0.250) showed a significant interaction. These analyses cannot rank modalities, establish superiority of any short-bout approach, or determine that study setting modifies the effect (44–49).

Evidence focused on sedentary interruption remains directly aligned with this review. Frequent brief walking or resistance bouts may be behaviorally accessible and may attenuate postprandial exposure. Protocols nevertheless ranged from four 1-min snacks/day to 60-min sessions, while Duvivier (2017) (34) included more than 2 h/day of added walking. The core-definition sensitivity retained a similar point estimate but was imprecise. The category interaction did not explain heterogeneity, indicating that protocol labels alone do not capture potentially important differences in dose, timing, comparator, monitoring window, or metabolic context.

Mechanistically, the findings are biologically plausible through two linked pathways: reducing prolonged skeletal-muscle inactivity and repeatedly recruiting contracting muscle during periods that would otherwise be sedentary. Skeletal muscle is central to postprandial glucose disposal, and recent mechanistic work emphasizes that muscle glucose uptake depends on coordinated delivery from the circulation, transendothelial transport, sarcolemmal transport, and intracellular metabolism (50). Brief repeated activity bouts may increase muscle blood flow, stimulate glucose transport, activate energy-sensing pathways, and shorten periods of very low contractile activity. These mechanisms help explain why short activity breaks may preferentially influence postprandial exposure or selected variability metrics. Nevertheless, this meta-analysis did not directly measure GLUT4 translocation, AMPK activation, mitochondrial function, endothelial function, or insulin sensitivity. Mechanistic interpretation should therefore remain explanatory rather than causal (51, 52).

CGM outcomes are not interchangeable. Mean glucose is a broad exposure summary, whereas MAGE, SD, CV, TIR, time above a threshold, and iAUC depend on monitoring duration, thresholds, meals, missing-data rules, and algorithms. The original pooled time-in-hyperglycemia result combined clinically different definitions; after stratification, estimates differed sharply between 22- and 24-h windows. Future trials should use consensus CGM definitions, prespecify core outcomes and analysis windows, and report paired contrasts with SEs or within-person correlations.

### 4.3 Limitations

This review has several limitations. Thirteen studies were eligible, samples ranged from 7 to 41, and most interventions lasted hours or days. Residual heterogeneity persisted despite sensitivity analyses, and the wide prediction interval spanned substantial benefit to possible harm. Dempsey (2017) (33) strongly influenced magnitude and heterogeneity; omitting it produced a smaller favorable estimate, but exclusion solely for influence would be *post-hoc*. The pooled effect therefore is not stable across settings. Evidence cannot determine long-term adherence, HbA1c, body weight, or complication risk.

Generalizability is also restricted. Several trials were tightly controlled laboratory experiments with standardized meals, several enrolled only or predominantly men, one enrolled only older women, and most excluded insulin users, people with unstable disease, severe complications, or exercise contraindications. Free-living or mixed-setting studies were internally consistent, whereas heterogeneity was concentrated among controlled laboratory studies; nevertheless, the exploratory setting interaction was non-significant and cannot establish transportability. Results should not be directly extrapolated to excluded populations or to sustained real-world implementation. Paired covariance was incompletely reported and required the primary assumed within-person correlation of *r* = 0.50 for several studies, although *r* = 0.25 and *r* = 0.75 analyses were nearly identical. Multi-arm selections were addressed by one-at-a-time replacement analyses. The exploratory Egger-type test was non-significant, but exactly 10 studies and high heterogeneity limit the ability to exclude small-study or missing-evidence bias.

### 4.4 Practical implications

Short-bout activity remains a plausible complementary option for adults with stable type 2 diabetes who have difficulty sustaining structured exercise. The point estimate may be clinically relevant, but the robust CI and prediction interval do not support presenting the strategy as established glucose-lowering therapy. It should not replace recommended structured exercise, pharmacotherapy, or individualized clinical management. People using insulin or secretagogues, those with cardiovascular or microvascular complications, and those with exercise limitations require individualized assessment and monitoring.

Future studies should use larger samples, adequate randomization, preregistration, and transparent trial reporting. They should harmonize core CGM outcomes, thresholds, and monitoring windows. Crossover reports should provide paired-difference SDs, SEs, or within-person correlations. Longer interventions should evaluate maintenance, TIR/TAR, hypoglycemia, adverse events, real-world adherence, and patient-important outcomes. These improvements are needed to determine the optimal type, dose, timing, and target population for short-bout activity strategies (53).

## 5 Conclusion

Short-bout accumulated physical activity may reduce mean glucose in adults with type 2 diabetes, but this low-certainty finding rests on 10 small crossover studies. Considerable heterogeneity, individual-study influence, and a prediction interval that included the null value (no effect) limit confidence that the pooled estimate applies in a new setting. Exploratory subgroup analyses do not establish superiority of any intervention modality. Evidence for other glycemic outcomes and safety remains uncertain. Larger preregistered trials with harmonized CGM outcomes, paired effects, and longer follow-up are needed.

## Data Availability

The original contributions presented in the study are included in the article/**Supplementary Material**. Further inquiries can be directed to the corresponding author.
