# Supplementary material for: Effects of short-bout accumulated physical activity and sedentary-break interventions on continuous glucose monitoring metrics in adults with type 2 diabetes: A systematic review and meta-analysis
Source: Front Endocrinol (Lausanne). 2026 Sep 11;17:1901259. doi: 10.3389/fendo.2026.1901259 (PMC13612268; doi:10.3389/fendo.2026.1901259)
Supplement: Supplementary file 1 [file Supplementaryfile1.docx]

**Supplementary Material**

All formal tables use a three-line format. Calculations retain full precision; displayed values use standard ROUND_HALF_UP formatting with trailing zeros removed.

**Supplementary Table S1. Population and trial-method characteristics**

| **Study** | **Country** | **n** | **Women/men** | **Age, years** | **Population** | **Design/setting** | **CGM window** | **Source** |
| --- | --- | --- | --- | --- | --- | --- | --- | --- |
| Babir 2026 | Canada | 31 | 21/10 | 58 (11) | 31; non-insulin-treated; inactive; HbA1c 6.6% | Randomized counterbalanced crossover; real world; standardized diet | 48 h per condition; 24 h washout | Article Table 1 and Methods |
| Blankenship 2019 | United States | 30 | 16/14 | 64 (8.2) | Sedentary; mean BMI 31.7 kg/m2; HbA1c 7.4% | Randomized crossover; free living; diet controlled | 7 d CGM; three 24 h experimental conditions | Article Table 2 and Methods |
| Dempsey 2017 | Australia | 24 | 10/14 | 62 (6) | Inactive; diet/metformin controlled; BMI 33.0 kg/m2 | Randomized crossover laboratory study; mixed models adjusted for period | 22 h CGM following 7 h laboratory condition | Article Table 1 and Methods |
| Duvivier 2017 | Netherlands | 19 | 6/13 | 63 (9) | Non-insulin-treated; BMI 30.5 kg/m2; HbA1c 6.7% | Blocked randomized crossover; free living | 4 d regimens; 24 h CGM on day 4 | Article Table 1 and Methods |
| Haxhi 2016 | Italy | 9 | 0/9 | 58.2 (6.6) | Sedentary men; BMI 30.2 kg/m2; oral therapy | Randomized crossover lunchtime exercise trial | 24 h CGM after lunch intervention | Article Table 1 and Methods |
| Homer 2021 | Australia | 24 | 11/13 | 62 (8) | Medically controlled; non-insulin; inactive | Randomized balanced-block crossover laboratory study | 22 h FGM after 7 h sitting condition | Abstract and Methods |
| Metcalfe 2018 | United Kingdom | 11 | 0/11 | 52 (6) | Men; BMI 29.7 kg/m2; HbA1c 7.0% | Randomized four-condition crossover; standardized diet | 24 h CGM after experimental condition | Abstract and Methods |
| van Dijk 2013 | Netherlands | 20 | 0/20 | 64 (1 SE) | Men; BMI 29.5 kg/m2; HbA1c 6.9% | Randomized three-condition crossover | Three 3 d periods; 24 h CGM on test day | Article Table 1 and Methods |
| Gillen 2012 | Canada | 7 | NR | 62 (3) | Adults with T2DM; BMI 30.5 kg/m2; HbA1c 6.9%; 6/7 used glucose-lowering medication | Fixed-order controlled within-person comparison; laboratory exercise with standardized diet | 24 h CGM after HIT and control days | Participants/Experimental Design |
| Myette-Côté 2018 | Canada | 11 | 7/4 | 64 (8) | Completers with T2DM; BMI 34.0 kg/m2; HbA1c 7.0%; non-insulin treated | Randomized three-condition crossover; provided diets and free-living postmeal walking | Four-day iPro2 CGM per condition | Table 2 and Methods |
| Terada 2016 | Canada | 10 | 2/8 | 60 (6) | Adults with T2DM; BMI 30.8 kg/m2; HbA1c 7.1% | Randomized five-condition controlled crossover; supervised laboratory exercise | 24 h iPro2 CGM; outcome-specific n=7-10 | Table 1 and Methods |
| de Hoogh 2025 | Netherlands | 41 | 19/22 | 62.3 (7.2) | Insulin-naive adults with T2DM; BMI 29.2 kg/m2; HbA1c 7.1%; lifestyle/metformin | Randomized repeated-measures study with eleven four-day periods; free living | Dexcom G6; eleven four-day self-monitoring periods | Table 1 and Methods 2.1-2.4 |
| Marcotte-Chénard 2024 | Canada | 14 | 14/0 | 70 (4) | Older women with T2DM; BMI 33.2 kg/m2; mean diabetes duration 10 years | Semi-randomized three-condition acute crossover; control fixed first | Dexcom G6; 24 h after each condition | Participants, Figure 3, Table 1 |

**Supplementary Table S2. Medication, CGM methods, randomization, washout, adherence, and sensor completeness**

| **Study** | **Medication** | **CGM/FGM device** | **Calibration** | **Randomization** | **Washout** | **Adherence** | **Sensor completeness** |
| --- | --- | --- | --- | --- | --- | --- | --- |
| Babir 2026 | Non-insulin; <=3 glucose-lowering drugs; most used glucose-lowering medication | FreeStyle Libre 2 | Factory calibrated | Counterbalanced; concealed envelopes; sequence generated independently and stratified by centre | 24 h; one participant did not complete the full washout | Self-report 4 (1) snacks/day; Fitbit available for a subset | 31 completed both conditions; outcome-specific n=29-31 |
| Blankenship 2019 | Glucose-lowering medications continued as prescribed | Medtronic iPro2 | Capillary glucose 3-4 times/day | Randomized counterbalanced crossover; sequence-generation/concealment details NR | One habitual-activity day | activPAL-confirmed; described as high compliance | Periods with <=80% CGM completeness excluded |
| Dempsey 2017 | Diet or metformin controlled; metformin 23/24 | Medtronic iPro2 with Enlite sensor | Six standardized fingersticks over 22 h | Third-party balanced block randomization, stratified by sex | 6-14 d | Laboratory-supervised activity and posture monitoring | 24/24 completed all conditions and 22 h monitoring |
| Duvivier 2017 | No insulin; oral glucose-lowering drugs 14/19 | Medtronic iPro2 Professional with Enlite sensor | Four capillary measurements/day | Blocked randomization with sealed opaque envelopes | >=10 d | Accelerometry and activity diaries | Last 24 h of each regimen; 288 CGM values/day |
| Haxhi 2016 | Metformin 4/9; metformin plus DPP-4 inhibitor 5/9 | Medtronic CGMS iPro | Capillary glucose four times/day | Randomized crossover; sequence/concealment details NR | >=1 week | Treadmill sessions supervised | 24 consecutive hours analysed; completeness criterion NR |
| Homer 2021 | One to three non-insulin antihyperglycaemic agents | Abbott FreeStyle Libre FGM | Factory calibrated; 1 h startup period | Third-party balanced block randomization | 6-14 d | Laboratory-supervised activity; lead-in adherence confirmed | Nine missing baseline observations caused by sensor replacement were substituted as prespecified |
| Metcalfe 2018 | No exogenous insulin; no more than two glucose-lowering medications | Medtronic iPro2 with Enlite sensor | Capillary measurements before meals and sleep | Randomized envelope method; concealment detail NR | >=5 d | All participants completed exercise sessions; pretrial activity checked | 11/11 completed all conditions; completeness criterion NR |
| van Dijk 2013 | Oral glucose-lowering medication continued | GlucoDay S | Capillary sampling used; exact calibration schedule NR | Randomized crossover; sequence/concealment details NR | >=1 week | Laboratory meals and activity periods supervised | 24 h CGM analysed; completeness criterion NR |
| Gillen 2012 | Six of seven used glucose-lowering medication; unchanged | Medtronic iPro CGM | NR | Not randomized: CTL always preceded HIT | Approximately 1 week | Exercise supervised; standardized dietary intake | 7/7 completed both measurement days |
| Myette-Côté 2018 | No exogenous insulin; varied oral/GLP-1 therapies | Medtronic iPro2 with Enlite sensor | Capillary calibration per device protocol | Computer-generated randomized condition order | 9-14 d | Actigraph monitoring confirmed postmeal walks; provided diets | 11/16 randomized completed all three periods; reasons documented |
| Terada 2016 | Usual glucose-lowering therapy; exact distribution in article | Medtronic iPro2 | Capillary calibration per device protocol | Random order of five conditions; concealment details NR | 48 h | Laboratory-supervised exercise and standardized meals | Outcome-specific n=7-10 because usable CGM/condition data varied |
| de Hoogh 2025 | Lifestyle and/or metformin; one participant withdrew after starting gliclazide | Dexcom G6 | Daily finger-prick calibration reported | Intervention-period order randomized per participant | >=1 week | Smartphone logs, activity/sleep monitoring, duplicated intervention periods | 42 started; 41 baseline; approximately 40 analysed; result-specific n not consistently locked |
| Marcotte-Chénard 2024 | Stable glucose-lowering medication; no insulin | Dexcom G6 | Factory calibrated | CON fixed first; HIIT10/HIIT4 order randomized with concealed allocation | 48 h between visits; 96 h where specified | Exercise supervised | 15 randomized; 14 had complete CGM for all conditions |

**Supplementary Table S3. Quantitative intervention boundaries and post hoc core-definition assessment**

| **Study** | **Main arm** | **Protocol** | **Bout min** | **Bouts/day** | **Active min/day** | **Break frequency** | **Standing min/day** | **Core eligible** | **Rule** | **Source** |
| --- | --- | --- | --- | --- | --- | --- | --- | --- | --- | --- |
| Babir 2026 | Exercise snacks | Four 1 min vigorous bodyweight bouts/day | 1 | 4 | 4 | Not a sedentary-break schedule | 0 | Yes | Exercise-snack rule | Article Methods, pp. 3-4 |
| Blankenship 2019 | Postmeal activity breaks | Four breaks every 30 min for 2 h after each meal; 12/day; group-matched total 20, 40, or 60 min/day | 1.67/3.33/5 | 12 | 20/40/60 | Every 30 min after meals | 0 | Yes | Sedentary-break rule | Article Methods, pp. 3-4 |
| Dempsey 2017 | Light walking breaks | 3 min light walking every 30 min during 7 h sitting | 3 | 12 | 36 | Every 30 min | 0 | Yes | Sedentary-break rule | Article Methods, p. 3 |
| Duvivier 2017 | Sit Less | Replaced about 4.7 h/day sitting with about 2.5 h standing and 2.2 h light walking | Distributed/NR | NR | 132 min walking | Free-living redistribution | 150 min | No | Active time >60 min/day; standing alone excluded | Article Methods, pp. 3-4 |
| Haxhi 2016 | SplitEx | 20 min brisk walking before lunch and 20 min 40 min after lunch | 20 | 2 | 40 | Around lunch | 0 | Yes | Accumulated postprandial walking rule | Article Methods, pp. 3-4 |
| Homer 2021 | SRA3 | 3 min simple resistance activities every 30 min during 7 h sitting | 3 | 12 | 36 | Every 30 min | 0 | Yes | Sedentary-break rule | Article Methods, p. 4 |
| Metcalfe 2018 | REHIT | Two 20 s all-out sprints within a 10 min session | 0.33 work bout | 2 | 10 min session; 0.67 min sprint work | Single post-breakfast session | 0 | Yes | Low-volume interval rule | Article Abstract and Methods |
| van Dijk 2013 | Activities of daily living | 15 min walking after breakfast, lunch, and dinner | 15 | 3 | 45 | After each main meal | 0 | Yes | Accumulated postprandial walking rule | Article Methods, p. 2 |
| Gillen 2012 | HIT | 10 x 60 s cycling at approximately 90% HRmax with 60 s recovery; warm-up/cool-down | 1 | 10 | 10 min high-intensity work; approximately 25 min complete session | Single session | 0 | No | Low-volume interval boundary; complete session >10 min | Exercise Protocol |
| Myette-Côté 2018 | LC+Ex | 15 min walking beginning approximately 30 min after breakfast, lunch and dinner for four days | 15 | 3 | 45 | After each main meal | 0 | Yes | Accumulated postprandial walking rule | Experimental protocol |
| Terada 2016 | HIIEfast | 15 cycles of 3 min at 40% plus 1 min at 100% VO2peak; 60 min complete session | 1 min work interval | 15 | 15 min high-intensity work; 60 min complete session | Single session | 0 | No | Wide-definition boundary; complete session >10 min | Methods 2.4 |
| de Hoogh 2025 | Active Day | 5 min moderate-to-intense activity every hour from 09:00 to 17:00; WaM alternative 3 x 15 min | 5 | 9 scheduled | 45 scheduled | Hourly | 0 | Yes | Sedentary-break rule; WaM also core eligible | Methods 2.3 |
| Marcotte-Chénard 2024 | HIIT10 | 10 x 1 min at 90% HRmax; complete session approximately 38 min | 1 | 10 | 10 min high-intensity work; 38 min complete session | Single session | 0 | No | Wide-definition boundary; complete session >10 min; HIIT4 ineligible | Exercise and control conditions |

Post hoc core intervention definition: active sedentary breaks or exercise snacks ≤10 min/bout, ≥2/day, and ≤60 active min/day; accumulated postprandial walking ≤20 min/bout, ≥2/day, and ≤60 active min/day; low-volume interval work bouts ≤1 min within a complete session ≤10 min. Standing alone did not qualify. NR, not reported.

**Supplementary Table S4. Study-by-outcome availability matrix**

| **Study** | **Mean glucose** | **MAGE** | **SD** | **CV** | **TIR** | **Hyperglycaemia time** | **iAUC** | **Safety** |
| --- | --- | --- | --- | --- | --- | --- | --- | --- |
| Babir 2026 | pooled | pooled | pooled | pooled | pooled | narrative | narrative | narrative |
| Blankenship 2019 | pooled | not reported | not reported | pooled | not reported | pooled | narrative | not reported |
| Dempsey 2017 | pooled | pooled | pooled | pooled | not reported | pooled | narrative | narrative |
| Duvivier 2017 | pooled | not reported | not reported | not reported | not reported | pooled | narrative | narrative |
| Haxhi 2016 | pooled | pooled | pooled | not reported | not reported | narrative | narrative | not reported |
| Homer 2021 | pooled | not reported | pooled | pooled | pooled | pooled | narrative | narrative |
| Metcalfe 2018 | pooled | pooled | narrative | not reported | not reported | narrative | narrative | narrative |
| van Dijk 2013 | non-extractable | not reported | not reported | not reported | not reported | pooled | narrative | not reported |
| Gillen 2012 | pooled | not reported | not reported | not reported | not reported | pooled | narrative | not reported |
| Myette-Côté 2018 | pooled | pooled | pooled | not reported | not reported | narrative | not reported | not reported |
| Terada 2016 | pooled | pooled | not reported | not reported | not reported | pooled | narrative | narrative |
| de Hoogh 2025 | non-extractable | non-extractable | not reported | non-extractable | non-extractable | non-extractable | not reported | narrative |
| Marcotte-Chénard 2024 | non-extractable | non-extractable | non-extractable | not reported | non-extractable | non-extractable | not reported | narrative |

Pooled, included in the specified synthesis; narrative, reported without quantitative pooling; duplicate, not entered twice; non-extractable, insufficient paired information; not reported, absent from the report. Not reported was never interpreted as zero.

**Supplementary Table S5. Hyperglycaemia thresholds, observation windows, and pooling decisions**

| **Study** | **Threshold** | **Window** | **Denominator/metric** | **Pooling decision** | **Reason** |
| --- | --- | --- | --- | --- | --- |
| Babir 2026 | >10.0 mmol/L | 48 h | Binary occurrence of TAR | No | Not a continuous exposure measure |
| Blankenship 2019 | >10 mmol/L | 24 h | Hours and percentage of 24 h | Yes: 24 h >10 stratum | Paired SE imputed at r=0.50 |
| Dempsey 2017 | >10 mmol/L | 22 h | Hours and percentage of 22 h | Yes: 22 h >10 stratum | Not converted to 24 h |
| Duvivier 2017 | >=10.0 mmol/L | 24 h | Minutes and percentage of 24 h | Yes: 24 h >10 stratum | Sit Less outside post hoc core definition |
| Haxhi 2016 | >150 mg/dL (~8.3 mmol/L) | 24 h | Percentage of 24 h | No; single-study result | Threshold differs materially |
| Homer 2021 | >10 mmol/L | 22 h | Hours and percentage of 22 h | Yes: 22 h >10 stratum | Not converted to 24 h |
| Metcalfe 2018 | >=9 mmol/L | 24 h | Minutes and percentage of 24 h | No; single-study result | Threshold differs materially |
| van Dijk 2013 | >10 mmol/L | 24 h | Minutes and percentage of 24 h | Yes: 24 h >10 stratum | SE derived from repeated-measures P=0.67 |
| Gillen 2012 | >=10 mmol/L | 24 h | Percentage of 24 h | Yes: 24 h >10 stratum | Threshold/window compatible; paired SE imputed at r=0.50 |
| Myette-Côté 2018 | >10 mmol/L | Four days | Percentage of four-day CGM time | No; structured narrative | Monitoring window differs from 24 h and 22 h strata |
| Terada 2016 | >10 mmol/L | 24 h | Minutes converted to percentage points of 24 h | Yes: 24 h >10 stratum | MD and CI divided by 14.4; original minutes retained in conversion log |
| de Hoogh 2025 | TAR-L1/TAR-L2 using consensus CGM ranges | Four days | Conditional model coefficients | No; non-extractable | No full-sample marginal effect/covariance |
| Marcotte-Chénard 2024 | >=8.3 and >=10 mmol/L | 24 h | Median [IQR] minutes | No; non-extractable | Threshold-specific medians/IQR and no paired SE |

Only compatible thresholds, observation windows, and denominators were combined. Twenty-two-hour results were not relabelled as actual 24-hour exposure.

**Supplementary Table S6. Study-level hypoglycaemia and adverse-event reporting**

| **Study** | **Outcome** | **Definition** | **Events** | **Interpretation** | **Source/note** |
| --- | --- | --- | --- | --- | --- |
| Babir 2026 | Hypoglycaemia/TBR | TBR <3.9 mmol/L dichotomized | ES 9/31; CON 10/31 had any TBR | No clear difference | Not a systematic adverse-event table |
| Blankenship 2019 | Adverse events/hypoglycaemia | Not reported | NR | Unknown | Do not code NR as zero |
| Dempsey 2017 | Hypoglycaemia | Glucose <3.9 mmol/L | 0 episodes in all conditions | Zero observed | Article Results, p. 6 |
| Duvivier 2017 | Hypoglycaemia | <=3.9 mmol/L plus symptoms | 0 periods in all regimens | Zero observed | Article Results, p. 5 |
| Haxhi 2016 | Adverse events/hypoglycaemia | Not reported | NR | Unknown | Do not code NR as zero |
| Homer 2021 | CGM-detected hypoglycaemia | <3.9 mmol/L | 5 participants: SIT 3; SRA3 1; SRA6 1; asymptomatic | No excess signal; one episode checked as sensor artefact | Article Discussion, p. 9 |
| Metcalfe 2018 | Adverse events | Symptoms plus CGM review | 1 subjective episode during HIIT; CGM 5-6 mmol/L; no other events | Not in primary REHIT arm | Article Results, p. 8 |
| van Dijk 2013 | Adverse events/hypoglycaemia | Not reported | NR | Unknown | Do not code NR as zero |
| Gillen 2012 | Adverse events/hypoglycaemia | Not reported | NR | Unknown | Do not code NR as zero |
| Myette-Côté 2018 | Adverse events | Not systematically reported | 5/16 did not complete: family reason, diet adherence (3), medication change (1) | No withdrawal was reported as exercise-related harm; formal safety remains unknown | CONSORT flow diagram |
| Terada 2016 | Adverse events/withdrawal | Narrative | One participant completed only one condition because of an injury unrelated to the study | No study-related harm signal, but systematic ascertainment NR | Results/participant flow |
| de Hoogh 2025 | Adverse events/withdrawal | Narrative | One withdrawal after starting gliclazide; exercise-related adverse events NR | Medication-related protocol withdrawal; other harms unknown | Results 3.1 |
| Marcotte-Chénard 2024 | Hypoglycaemia/other events | CGM <3.9 mmol/L plus participant flow | 8/14 had no hypoglycaemia in any condition; six had brief episodes; severe dizziness occurred before randomization/was not clearly attributable | No clear exercise-related excess; ascertainment and attribution limited | Results, Figure 3, Table 2 |

Zero events and non-reporting were kept distinct. Safety was not quantitatively pooled because definitions and ascertainment were inconsistent.

**Supplementary Table S7. Outcome-specific RoB 2 judgments for crossover trials**

| **Study** | **Result/time point** | **D1** | **Crossover period/carryover** | **D2** | **D3** | **D4** | **D5** | **Overall** | **Basis** |
| --- | --- | --- | --- | --- | --- | --- | --- | --- | --- |
| Babir 2026 | Overall mean glucose, 48 h | Low | Low | Low | Low | Low | Low | Low | Concealed counterbalanced sequence; objective CGM; registered trial; non-blinding alone did not trigger D2 concerns |
| Blankenship 2019 | Overall mean glucose, 24 h | Some concerns | Some concerns | Low | Low | Low | Some concerns | Some concerns | Random-sequence/concealment and prespecified analysis details insufficient |
| Dempsey 2017 | Overall mean glucose, 22 h | Low | Low | Low | Low | Low | Low | Low | Randomized sequence; period adjusted mixed model; objective CGM; registered |
| Duvivier 2017 | Overall mean glucose, 24 h | Low | Low | Low | Low | Low | Low | Low | Blocked randomization with sealed opaque envelopes; objective CGM |
| Haxhi 2016 | Overall mean glucose, 24 h | Some concerns | Some concerns | Low | Low | Low | Some concerns | Some concerns | Sequence concealment/carryover and prospective analysis details insufficient |
| Homer 2021 | Overall mean glucose, 22 h | Low | Low | Low | Low | Low | Low | Low | Third-party balanced block randomization; 6-14 d washout; 24/24 completed all conditions; the nine substituted pre-breakfast baseline readings applied to meal-specific iAUC rather than the 22-h mean-glucose result. |
| Metcalfe 2018 | Overall mean glucose, 24 h | Low | Low | Low | Low | Low | Low | Low | Registered randomized four-condition crossover; envelope-method condition order; >=5 d washout; 11/11 completed all conditions; objective CGM. |
| van Dijk 2013 | Time >10 mmol/L, 24 h | Some concerns | Some concerns | Low | Low | Low | Low | Some concerns | Sequence/concealment and carryover reporting limited; objective CGM |
| Dempsey 2017 | Hypoglycaemia, 22 h | Low | Low | Low | Low | Low | Some concerns | Some concerns | Zero events reported, but safety was not the primary registered result |
| Duvivier 2017 | Hypoglycaemia, 24 h | Low | Low | Low | Low | Low | Some concerns | Some concerns | Zero periods reported; safety ascertainment less detailed than efficacy ascertainment |
| Homer 2021 | Hypoglycaemia, 22 h | Low | Low | Low | Some concerns | Some concerns | Some concerns | Some concerns | FGM false-low concern and incomplete confirmation of detected episodes |
| Metcalfe 2018 | Adverse events, 24 h | Some concerns | Low | Low | Low | Some concerns | Some concerns | Some concerns | Events were narratively ascertained in a small unblinded study |
| Gillen 2012 | Overall mean glucose, 24 h | High | High | Low | Low | Low | Some concerns | High | CTL was always measured before HIT, so treatment is confounded with period/order despite objective CGM |
| Gillen 2012 | Time >=10 mmol/L, 24 h | High | High | Low | Low | Low | Some concerns | High | Fixed order; outcome objectively measured but selection from prior cohort and no prospective analysis plan |
| Myette-Côté 2018 | Overall mean glucose, four days | Low | Low | Low | Some concerns | Low | Some concerns | Some concerns | Randomized order and adequate washout; 5/16 did not complete and pairwise Fisher-LSD reporting was not accompanied by a prespecified paired SE |
| Myette-Côté 2018 | Safety, four days | Low | Low | Low | Some concerns | Some concerns | Some concerns | Some concerns | Withdrawals documented but adverse events were not systematically reported |
| Terada 2016 | Overall mean glucose, 24 h | Some concerns | Low | Low | Some concerns | Low | Some concerns | Some concerns | Randomized crossover with washout; concealment and outcome-specific missing CGM data/analysis plan incompletely reported |
| Terada 2016 | Safety, 24 h | Some concerns | Low | Low | Some concerns | Some concerns | Some concerns | Some concerns | One unrelated injury was reported; systematic harm ascertainment was limited |
| de Hoogh 2025 | Mean glucose, four days | Low | Low | Low | Some concerns | Low | Some concerns | Some concerns | Randomized repeated periods and objective CGM; result-specific n and full-sample marginal estimand not fully reported; exploratory interaction model |
| de Hoogh 2025 | Safety, four days | Low | Low | Low | Some concerns | Some concerns | Some concerns | Some concerns | One medication-related withdrawal; exercise harms not systematically tabulated |
| Marcotte-Chénard 2024 | Mean glucose, 24 h | High | High | Low | Low | Low | Some concerns | High | Control condition was fixed first; treatment is confounded with period/order, and only median/IQR results were available |
| Marcotte-Chénard 2024 | Safety, 24 h | High | High | Low | Some concerns | Some concerns | Some concerns | High | Fixed control-first order and limited event ascertainment/attribution |

Non-blinding was not by itself grounds for a D2 downgrade. Judgments concern the specified result and time point.

**Supplementary Table S8. GRADE evidence profile**

| **Outcome** | **Studies (participants)** | **Effect (95% CI)** | **Risk of bias** | **Inconsistency** | **Indirectness** | **Imprecision** | **Publication bias** | **Certainty** | **Decision basis** |
| --- | --- | --- | --- | --- | --- | --- | --- | --- | --- |
| Overall mean glucose | 10 (175) | -0.71 (-1.28 to -0.13) | Not serious | Serious | Not serious | Serious | Not serious; exploratory Egger-type P=0.488 | Low | MID=0.5 mmol/L; OIS=45 and observed n=175. The CI includes both a clinically important and a smaller benefit. High-risk Gillen carried 9.1% weight and its leave-one-out result remained concordant; objective CGM and no dominant high-risk result support no RoB downgrade. Prediction interval crosses the null. |
| Glucose CV | 3 (85) | 0.69 (-3.86 to 5.23) | Not serious | Serious | Not serious | Serious | Undetected; k<10, no specific signal | Low | MID=1.14 percentage points; OIS=209. No high-risk result dominated; objective CGM. Wide, heterogeneous estimates warrant inconsistency and imprecision downgrades. |
| MAGE | 6 (95) | -0.63 (-1.25 to -0.02) | Not serious | Serious | Not serious | Serious | Undetected; k<10, no specific signal | Low | MID=0.34 mmol/L; OIS=199. No pooled result was high risk overall; objective CGM. CI spans trivial to important benefit and heterogeneity is substantial. |
| Glucose SD | 4 (75) | -0.14 (-0.44 to 0.16) | Not serious | Serious | Not serious | Serious | Undetected; k<10, no specific signal | Low | MID=0.1 mmol/L; OIS=189. No high-risk result dominated; objective CGM. CI crosses the null and clinical threshold; inconsistency is substantial. |
| Time in range | 2 (55) | 1.88 (-2.07 to 5.82) | Not serious | Not serious | Not serious | Serious | Undetected; k<10 | Moderate | MID=5 percentage points; OIS=111. Only two studies; CI includes no effect and a potentially important increase. HKSJ is shown as sensitivity because k=2. |
| Time >10 mmol/L (24 h) | 5 (85) | -7.26 (-11.09 to -3.43) | Not serious | Not serious | Not serious | Serious | Undetected; k<10 | Moderate | MID=5 percentage points; OIS=280. Threshold, 24-h window and percentage-point denominator were harmonized; Terada minutes were transparently converted. CI spans smaller and important benefit. |
| Time >10 mmol/L (22 h) | 2 (48) | -20.83 (-240.3 to 198.64) | Not serious | Very serious | Not serious | Very serious | Undetected; k<10 | Very low | MID=5 percentage points; OIS=114. Two highly inconsistent studies and an extremely wide interval; 22 h was not rescaled to 24 h. |
| Safety | 13 studies (inconsistent reporting) | Not pooled | Serious | Not applicable | Serious | Very serious | Undetected | Very low | Any clinically important increase in harm was the threshold. Safety was rarely prespecified or systematically ascertained; NR was not treated as zero. |

The search update is complete. Certainty was judged from RoB, inconsistency, indirectness, clinical thresholds, OIS, and missing-evidence considerations; statistical significance alone was not a downgrading rule.

**Supplementary Table S9. Optimal information size calculations**

| **Outcome** | **Formula** | **Representative paired SD** | **Clinical threshold** | **Required n** | **Observed n** | **OIS status** | **Note** |
| --- | --- | --- | --- | --- | --- | --- | --- |
| Overall mean glucose | Two-sided alpha=0.05; power=80%; paired crossover formula | 1.1922 | 0.5 | 45 | 175 | Met | Imprecision also judged from CI versus the clinical threshold, not statistical significance |
| Glucose CV | Two-sided alpha=0.05; power=80%; paired crossover formula | 5.8788 | 1.14 | 209 | 85 | Not met | Imprecision also judged from CI versus the clinical threshold, not statistical significance |
| MAGE | Two-sided alpha=0.05; power=80%; paired crossover formula | 1.7091 | 0.34 | 199 | 95 | Not met | Imprecision also judged from CI versus the clinical threshold, not statistical significance |
| Glucose SD | Two-sided alpha=0.05; power=80%; paired crossover formula | 0.4899 | 0.1 | 189 | 75 | Not met | Imprecision also judged from CI versus the clinical threshold, not statistical significance |
| Time in range | Two-sided alpha=0.05; power=80%; paired crossover formula | 18.7473 | 5 | 111 | 55 | Not met | Imprecision also judged from CI versus the clinical threshold, not statistical significance |
| Time >10 mmol/L (24 h) | Two-sided alpha=0.05; power=80%; paired crossover formula | 29.8469 | 5 | 280 | 85 | Not met | Imprecision also judged from CI versus the clinical threshold, not statistical significance |
| Time >10 mmol/L (22 h) | Two-sided alpha=0.05; power=80%; paired crossover formula | 19.0259 | 5 | 114 | 48 | Not met | Imprecision also judged from CI versus the clinical threshold, not statistical significance |

**Supplementary Table S10. Result-level RoB 2 judgments for key secondary and safety outcomes**

| **Study** | **Specific result/time point** | **D1** | **Crossover period/carryover** | **D2** | **D3** | **D4** | **D5** | **Overall** | **Outcome-specific basis** |
| --- | --- | --- | --- | --- | --- | --- | --- | --- | --- |
| Babir 2026 | MAGE (48 h) | Low | Low | Low | Low | Low | Low | Low | No additional outcome-specific concern beyond the study-level judgment. |
| Babir 2026 | Glucose SD (48 h) | Low | Low | Low | Low | Low | Low | Low | No additional outcome-specific concern beyond the study-level judgment. |
| Babir 2026 | Glucose CV (48 h) | Low | Low | Low | Low | Low | Low | Low | No additional outcome-specific concern beyond the study-level judgment. |
| Babir 2026 | TIR/TAR/TBR (48 h) | Low | Low | Low | Low | Low | Low | Low | No additional outcome-specific concern beyond the study-level judgment. |
| Babir 2026 | Safety/TBR (48 h) | Low | Low | Low | Low | Low | Some concerns | Some concerns | Safety was not consistently prespecified or systematically ascertained; non-reporting was not coded as zero events. |
| Blankenship 2019 | Glucose CV (24 h) | Some concerns | Some concerns | Low | Low | Low | Some concerns | Some concerns | No additional outcome-specific concern beyond the study-level judgment. |
| Blankenship 2019 | Time >10 mmol/L (24 h) | Some concerns | Some concerns | Low | Low | Low | Some concerns | Some concerns | No additional outcome-specific concern beyond the study-level judgment. |
| Blankenship 2019 | Safety reporting (24 h) | Some concerns | Some concerns | Low | Low | Low | Some concerns | Some concerns | Safety was not consistently prespecified or systematically ascertained; non-reporting was not coded as zero events. |
| Dempsey 2017 | MAGE (22 h) | Low | Low | Low | Low | Low | Low | Low | No additional outcome-specific concern beyond the study-level judgment. |
| Dempsey 2017 | Glucose SD (22 h) | Low | Low | Low | Low | Low | Low | Low | No additional outcome-specific concern beyond the study-level judgment. |
| Dempsey 2017 | Glucose CV (22 h) | Low | Low | Low | Low | Low | Low | Low | No additional outcome-specific concern beyond the study-level judgment. |
| Dempsey 2017 | Time >10 mmol/L (22 h) | Low | Low | Low | Low | Low | Low | Low | No additional outcome-specific concern beyond the study-level judgment. |
| Dempsey 2017 | Safety/hypoglycaemia (22 h) | Low | Low | Low | Low | Low | Some concerns | Some concerns | Safety was not consistently prespecified or systematically ascertained; non-reporting was not coded as zero events. |
| Duvivier 2017 | Time >=10 mmol/L (24 h) | Low | Low | Low | Low | Low | Low | Low | No additional outcome-specific concern beyond the study-level judgment. |
| Duvivier 2017 | Safety/hypoglycaemia (24 h) | Low | Low | Low | Low | Low | Some concerns | Some concerns | Safety was not consistently prespecified or systematically ascertained; non-reporting was not coded as zero events. |
| Haxhi 2016 | MAGE (24 h) | Some concerns | Some concerns | Low | Low | Low | Some concerns | Some concerns | No additional outcome-specific concern beyond the study-level judgment. |
| Haxhi 2016 | Glucose SD (24 h) | Some concerns | Some concerns | Low | Low | Low | Some concerns | Some concerns | No additional outcome-specific concern beyond the study-level judgment. |
| Haxhi 2016 | Time >8.3 mmol/L (24 h) | Some concerns | Some concerns | Low | Low | Low | Some concerns | Some concerns | No additional outcome-specific concern beyond the study-level judgment. |
| Haxhi 2016 | Safety reporting (24 h) | Some concerns | Some concerns | Low | Low | Low | Some concerns | Some concerns | Safety was not consistently prespecified or systematically ascertained; non-reporting was not coded as zero events. |
| Homer 2021 | Glucose SD (22 h) | Low | Low | Low | Low | Low | Low | Low | Third-party balanced block randomization and 6-14 d washout; 24/24 completed all conditions. Nine missing pre-breakfast baseline readings were handled for meal-specific iAUC and did not constitute missing data for the named 22-h summary result. |
| Homer 2021 | Glucose CV (22 h) | Low | Low | Low | Low | Low | Low | Low | Third-party balanced block randomization and 6-14 d washout; 24/24 completed all conditions. Nine missing pre-breakfast baseline readings were handled for meal-specific iAUC and did not constitute missing data for the named 22-h summary result. |
| Homer 2021 | TIR (22 h) | Low | Low | Low | Low | Low | Low | Low | Third-party balanced block randomization and 6-14 d washout; 24/24 completed all conditions. Nine missing pre-breakfast baseline readings were handled for meal-specific iAUC and did not constitute missing data for the named 22-h summary result. |
| Homer 2021 | Time >10 mmol/L (22 h) | Low | Low | Low | Low | Low | Low | Low | Third-party balanced block randomization and 6-14 d washout; 24/24 completed all conditions. Nine missing pre-breakfast baseline readings were handled for meal-specific iAUC and did not constitute missing data for the named 22-h summary result. |
| Homer 2021 | Safety/hypoglycaemia (22 h) | Low | Low | Low | Some concerns | Low | Some concerns | Some concerns | Safety was not consistently prespecified or systematically ascertained; non-reporting was not coded as zero events. |
| Metcalfe 2018 | MAGE (24 h) | Low | Low | Low | Low | Low | Low | Low | Registered randomized four-condition crossover; envelope-method condition order; >=5 d washout; 11/11 completed all conditions; objective CGM and prespecified CGM outcomes. |
| Metcalfe 2018 | Glucose SD (24 h) | Low | Low | Low | Low | Low | Low | Low | Registered randomized four-condition crossover; envelope-method condition order; >=5 d washout; 11/11 completed all conditions; objective CGM and prespecified CGM outcomes. |
| Metcalfe 2018 | Time >=9 mmol/L (24 h) | Low | Low | Low | Low | Low | Low | Low | Registered randomized four-condition crossover; envelope-method condition order; >=5 d washout; 11/11 completed all conditions; objective CGM and prespecified CGM outcomes. |
| Metcalfe 2018 | Safety/adverse events (24 h) | Low | Low | Low | Low | Low | Some concerns | Some concerns | Safety was not consistently prespecified or systematically ascertained; non-reporting was not coded as zero events. |
| van Dijk 2013 | Time >10 mmol/L (24 h) | Some concerns | Some concerns | Low | Low | Low | Low | Some concerns | No additional outcome-specific concern beyond the study-level judgment. |
| van Dijk 2013 | Safety reporting (24 h) | Some concerns | Some concerns | Low | Low | Low | Some concerns | Some concerns | Safety was not consistently prespecified or systematically ascertained; non-reporting was not coded as zero events. |
| Gillen 2012 | Time >=10 mmol/L (24 h) | High | High | Low | Low | Low | Some concerns | High | Fixed CTL-first/HIT-second order confounds intervention with period. |
| Gillen 2012 | Postmeal peak/AUC (24 h) | High | High | Low | Low | Low | Some concerns | High | Fixed CTL-first/HIT-second order confounds intervention with period. |
| Gillen 2012 | Postmeal peak safety | High | High | Low | Some concerns | Some concerns | Some concerns | High | Fixed CTL-first/HIT-second order confounds intervention with period. Safety was not consistently prespecified or systematically ascertained. |
| Myette-Côté 2018 | MAGE/SD/CONGA (four days) | Low | Low | Low | Some concerns | Low | Some concerns | Some concerns | Randomized crossover; attrition and pairwise reporting introduce some concerns. |
| Myette-Côté 2018 | Time >10 mmol/L (four days) | Low | Low | Low | Some concerns | Low | Some concerns | Some concerns | Randomized crossover; attrition and pairwise reporting introduce some concerns. |
| Myette-Côté 2018 | Time >10 mmol safety | Low | Low | Low | Some concerns | Some concerns | Some concerns | Some concerns | Randomized crossover; attrition and pairwise reporting introduce some concerns. Safety was not consistently prespecified or systematically ascertained. |
| Terada 2016 | MAGE (24 h) | Some concerns | Low | Low | Some concerns | Low | Some concerns | Some concerns | Randomized five-period crossover; outcome-specific missing data and prospective selection details limited. |
| Terada 2016 | Time >10 mmol/L (24 h) | Some concerns | Low | Low | Some concerns | Low | Some concerns | Some concerns | Randomized five-period crossover; outcome-specific missing data and prospective selection details limited. |
| Terada 2016 | Postmeal iAUC (24 h) | Some concerns | Low | Low | Some concerns | Low | Some concerns | Some concerns | Randomized five-period crossover; outcome-specific missing data and prospective selection details limited. |
| Terada 2016 | Postmeal iAUC safety | Some concerns | Low | Low | Some concerns | Some concerns | Some concerns | Some concerns | Randomized five-period crossover; outcome-specific missing data and prospective selection details limited. Safety was not consistently prespecified or systematically ascertained. |
| de Hoogh 2025 | MAGE/CV/TIR/TAR (four days) | Low | Low | Low | Some concerns | Low | Some concerns | Some concerns | Objective CGM and randomized intervention order; conditional exploratory model and result-specific n limit reporting. |
| de Hoogh 2025 | MAGE safety | Low | Low | Low | Some concerns | Some concerns | Some concerns | Some concerns | Objective CGM and randomized intervention order; conditional exploratory model and result-specific n limit reporting. Safety was not consistently prespecified or systematically ascertained. |
| Marcotte-Chénard 2024 | MAGE/SD/TIR/TAR/TBR (24 h) | High | High | Low | Low | Low | Some concerns | High | Control was fixed first, creating period/order confounding. |
| Marcotte-Chénard 2024 | MAGE safety | High | High | Low | Some concerns | Some concerns | Some concerns | High | Control was fixed first, creating period/order confounding. Safety was not consistently prespecified or systematically ascertained. |

D1, randomization; S, period/carryover; D2, deviations from intended interventions; D3, missing outcome data; D4, outcome measurement; D5, selection of the reported result. Primary mean-glucose judgments are presented in the main traffic-light figure and Supplementary Tables S7 and S11.

**Supplementary Table S11. Crossover RoB 2 signaling-question responses for the primary CGM result**

| **Study** | **Result/time point** | **Domain** | **Signalling question** | **Response** | **Supporting information** | **Domain/overall link** |
| --- | --- | --- | --- | --- | --- | --- |
| Babir 2026 | Primary CGM result; 22-48 h as reported | D1 | 1.1 Was the allocation sequence random? | Yes | Registered trial; counterbalanced concealed sequence; one incomplete washout was disclosed; paired adjusted contrast reported. | Low |
| Babir 2026 | Primary CGM result; 22-48 h as reported | D1 | 1.2 Was the allocation sequence concealed until enrolment and assignment? | Yes | Registered trial; counterbalanced concealed sequence; one incomplete washout was disclosed; paired adjusted contrast reported. | Low |
| Babir 2026 | Primary CGM result; 22-48 h as reported | D1 | 1.3 Did baseline or period-start differences suggest a problem with randomization? | Probably no | Registered trial; counterbalanced concealed sequence; one incomplete washout was disclosed; paired adjusted contrast reported. | Low |
| Babir 2026 | Primary CGM result; 22-48 h as reported | Period/carryover | S1 Was the crossover design suitable for this acute outcome? | Yes | Registered trial; counterbalanced concealed sequence; one incomplete washout was disclosed; paired adjusted contrast reported. | Low |
| Babir 2026 | Primary CGM result; 22-48 h as reported | Period/carryover | S2 Was the washout sufficient to make important carryover unlikely? | Probably yes | Registered trial; counterbalanced concealed sequence; one incomplete washout was disclosed; paired adjusted contrast reported. | Low |
| Babir 2026 | Primary CGM result; 22-48 h as reported | Period/carryover | S3 Were period/order effects controlled or shown to be negligible? | Yes | Registered trial; counterbalanced concealed sequence; one incomplete washout was disclosed; paired adjusted contrast reported. | Low |
| Babir 2026 | Primary CGM result; 22-48 h as reported | D2 | 2.1 Were participants aware of their assigned intervention? | Yes | Registered trial; counterbalanced concealed sequence; one incomplete washout was disclosed; paired adjusted contrast reported. | Low |
| Babir 2026 | Primary CGM result; 22-48 h as reported | D2 | 2.2 Were intervention providers aware of assigned intervention? | Yes | Registered trial; counterbalanced concealed sequence; one incomplete washout was disclosed; paired adjusted contrast reported. | Low |
| Babir 2026 | Primary CGM result; 22-48 h as reported | D2 | 2.3 Were there trial-context deviations beyond the intended protocol? | No | Registered trial; counterbalanced concealed sequence; one incomplete washout was disclosed; paired adjusted contrast reported. | Low |
| Babir 2026 | Primary CGM result; 22-48 h as reported | D2 | 2.4 Were any deviations unbalanced and likely to affect the result? | Not applicable | Registered trial; counterbalanced concealed sequence; one incomplete washout was disclosed; paired adjusted contrast reported. | Low |
| Babir 2026 | Primary CGM result; 22-48 h as reported | D2 | 2.5 Was an appropriate assigned-intervention analysis used? | Yes | Registered trial; counterbalanced concealed sequence; one incomplete washout was disclosed; paired adjusted contrast reported. | Low |
| Babir 2026 | Primary CGM result; 22-48 h as reported | D3 | 3.1 Were outcome data available for all or nearly all randomized participants/periods? | Probably yes | Registered trial; counterbalanced concealed sequence; one incomplete washout was disclosed; paired adjusted contrast reported. | Low |
| Babir 2026 | Primary CGM result; 22-48 h as reported | D3 | 3.2 If data were missing, was bias from missingness unlikely? | Not applicable | Registered trial; counterbalanced concealed sequence; one incomplete washout was disclosed; paired adjusted contrast reported. | Low |
| Babir 2026 | Primary CGM result; 22-48 h as reported | D4 | 4.1 Was the CGM/FGM measurement method appropriate? | Yes | Registered trial; counterbalanced concealed sequence; one incomplete washout was disclosed; paired adjusted contrast reported. | Low |
| Babir 2026 | Primary CGM result; 22-48 h as reported | D4 | 4.2 Could measurement or ascertainment differ between conditions? | No | Registered trial; counterbalanced concealed sequence; one incomplete washout was disclosed; paired adjusted contrast reported. | Low |
| Babir 2026 | Primary CGM result; 22-48 h as reported | D4 | 4.3 Could awareness of condition influence the recorded outcome? | No | Registered trial; counterbalanced concealed sequence; one incomplete washout was disclosed; paired adjusted contrast reported. | Low |
| Babir 2026 | Primary CGM result; 22-48 h as reported | D5 | 5.1 Was the reported analysis selected according to a prespecified plan? | Yes | Registered trial; counterbalanced concealed sequence; one incomplete washout was disclosed; paired adjusted contrast reported. | Low |
| Babir 2026 | Primary CGM result; 22-48 h as reported | D5 | 5.2 Was selection from multiple eligible outcome measurements unlikely? | Probably yes | Registered trial; counterbalanced concealed sequence; one incomplete washout was disclosed; paired adjusted contrast reported. | Low |
| Babir 2026 | Primary CGM result; 22-48 h as reported | D5 | 5.3 Was selection from multiple eligible analyses unlikely? | Probably yes | Registered trial; counterbalanced concealed sequence; one incomplete washout was disclosed; paired adjusted contrast reported. | Low |
| Blankenship 2019 | Primary CGM result; 22-48 h as reported | D1 | 1.1 Was the allocation sequence random? | Probably yes | Randomized counterbalanced crossover, but concealment and prospective analysis details were insufficient. | Some concerns |
| Blankenship 2019 | Primary CGM result; 22-48 h as reported | D1 | 1.2 Was the allocation sequence concealed until enrolment and assignment? | No information | Randomized counterbalanced crossover, but concealment and prospective analysis details were insufficient. | Some concerns |
| Blankenship 2019 | Primary CGM result; 22-48 h as reported | D1 | 1.3 Did baseline or period-start differences suggest a problem with randomization? | Probably no | Randomized counterbalanced crossover, but concealment and prospective analysis details were insufficient. | Some concerns |
| Blankenship 2019 | Primary CGM result; 22-48 h as reported | Period/carryover | S1 Was the crossover design suitable for this acute outcome? | Yes | Randomized counterbalanced crossover, but concealment and prospective analysis details were insufficient. | Some concerns |
| Blankenship 2019 | Primary CGM result; 22-48 h as reported | Period/carryover | S2 Was the washout sufficient to make important carryover unlikely? | Probably yes | Randomized counterbalanced crossover, but concealment and prospective analysis details were insufficient. | Some concerns |
| Blankenship 2019 | Primary CGM result; 22-48 h as reported | Period/carryover | S3 Were period/order effects controlled or shown to be negligible? | No information | Randomized counterbalanced crossover, but concealment and prospective analysis details were insufficient. | Some concerns |
| Blankenship 2019 | Primary CGM result; 22-48 h as reported | D2 | 2.1 Were participants aware of their assigned intervention? | Yes | Randomized counterbalanced crossover, but concealment and prospective analysis details were insufficient. | Some concerns |
| Blankenship 2019 | Primary CGM result; 22-48 h as reported | D2 | 2.2 Were intervention providers aware of assigned intervention? | Yes | Randomized counterbalanced crossover, but concealment and prospective analysis details were insufficient. | Some concerns |
| Blankenship 2019 | Primary CGM result; 22-48 h as reported | D2 | 2.3 Were there trial-context deviations beyond the intended protocol? | No | Randomized counterbalanced crossover, but concealment and prospective analysis details were insufficient. | Some concerns |
| Blankenship 2019 | Primary CGM result; 22-48 h as reported | D2 | 2.4 Were any deviations unbalanced and likely to affect the result? | Not applicable | Randomized counterbalanced crossover, but concealment and prospective analysis details were insufficient. | Some concerns |
| Blankenship 2019 | Primary CGM result; 22-48 h as reported | D2 | 2.5 Was an appropriate assigned-intervention analysis used? | Yes | Randomized counterbalanced crossover, but concealment and prospective analysis details were insufficient. | Some concerns |
| Blankenship 2019 | Primary CGM result; 22-48 h as reported | D3 | 3.1 Were outcome data available for all or nearly all randomized participants/periods? | Probably yes | Randomized counterbalanced crossover, but concealment and prospective analysis details were insufficient. | Some concerns |
| Blankenship 2019 | Primary CGM result; 22-48 h as reported | D3 | 3.2 If data were missing, was bias from missingness unlikely? | Not applicable | Randomized counterbalanced crossover, but concealment and prospective analysis details were insufficient. | Some concerns |
| Blankenship 2019 | Primary CGM result; 22-48 h as reported | D4 | 4.1 Was the CGM/FGM measurement method appropriate? | Yes | Randomized counterbalanced crossover, but concealment and prospective analysis details were insufficient. | Some concerns |
| Blankenship 2019 | Primary CGM result; 22-48 h as reported | D4 | 4.2 Could measurement or ascertainment differ between conditions? | No | Randomized counterbalanced crossover, but concealment and prospective analysis details were insufficient. | Some concerns |
| Blankenship 2019 | Primary CGM result; 22-48 h as reported | D4 | 4.3 Could awareness of condition influence the recorded outcome? | No | Randomized counterbalanced crossover, but concealment and prospective analysis details were insufficient. | Some concerns |
| Blankenship 2019 | Primary CGM result; 22-48 h as reported | D5 | 5.1 Was the reported analysis selected according to a prespecified plan? | Probably no | Randomized counterbalanced crossover, but concealment and prospective analysis details were insufficient. | Some concerns |
| Blankenship 2019 | Primary CGM result; 22-48 h as reported | D5 | 5.2 Was selection from multiple eligible outcome measurements unlikely? | No information | Randomized counterbalanced crossover, but concealment and prospective analysis details were insufficient. | Some concerns |
| Blankenship 2019 | Primary CGM result; 22-48 h as reported | D5 | 5.3 Was selection from multiple eligible analyses unlikely? | No information | Randomized counterbalanced crossover, but concealment and prospective analysis details were insufficient. | Some concerns |
| Dempsey 2017 | Primary CGM result; 22-48 h as reported | D1 | 1.1 Was the allocation sequence random? | Yes | Third-party balanced block randomization; 6-14 d washout; mixed model adjusted for period. | Low |
| Dempsey 2017 | Primary CGM result; 22-48 h as reported | D1 | 1.2 Was the allocation sequence concealed until enrolment and assignment? | Yes | Third-party balanced block randomization; 6-14 d washout; mixed model adjusted for period. | Low |
| Dempsey 2017 | Primary CGM result; 22-48 h as reported | D1 | 1.3 Did baseline or period-start differences suggest a problem with randomization? | Probably no | Third-party balanced block randomization; 6-14 d washout; mixed model adjusted for period. | Low |
| Dempsey 2017 | Primary CGM result; 22-48 h as reported | Period/carryover | S1 Was the crossover design suitable for this acute outcome? | Yes | Third-party balanced block randomization; 6-14 d washout; mixed model adjusted for period. | Low |
| Dempsey 2017 | Primary CGM result; 22-48 h as reported | Period/carryover | S2 Was the washout sufficient to make important carryover unlikely? | Yes | Third-party balanced block randomization; 6-14 d washout; mixed model adjusted for period. | Low |
| Dempsey 2017 | Primary CGM result; 22-48 h as reported | Period/carryover | S3 Were period/order effects controlled or shown to be negligible? | Yes | Third-party balanced block randomization; 6-14 d washout; mixed model adjusted for period. | Low |
| Dempsey 2017 | Primary CGM result; 22-48 h as reported | D2 | 2.1 Were participants aware of their assigned intervention? | Yes | Third-party balanced block randomization; 6-14 d washout; mixed model adjusted for period. | Low |
| Dempsey 2017 | Primary CGM result; 22-48 h as reported | D2 | 2.2 Were intervention providers aware of assigned intervention? | Yes | Third-party balanced block randomization; 6-14 d washout; mixed model adjusted for period. | Low |
| Dempsey 2017 | Primary CGM result; 22-48 h as reported | D2 | 2.3 Were there trial-context deviations beyond the intended protocol? | No | Third-party balanced block randomization; 6-14 d washout; mixed model adjusted for period. | Low |
| Dempsey 2017 | Primary CGM result; 22-48 h as reported | D2 | 2.4 Were any deviations unbalanced and likely to affect the result? | Not applicable | Third-party balanced block randomization; 6-14 d washout; mixed model adjusted for period. | Low |
| Dempsey 2017 | Primary CGM result; 22-48 h as reported | D2 | 2.5 Was an appropriate assigned-intervention analysis used? | Yes | Third-party balanced block randomization; 6-14 d washout; mixed model adjusted for period. | Low |
| Dempsey 2017 | Primary CGM result; 22-48 h as reported | D3 | 3.1 Were outcome data available for all or nearly all randomized participants/periods? | Probably yes | Third-party balanced block randomization; 6-14 d washout; mixed model adjusted for period. | Low |
| Dempsey 2017 | Primary CGM result; 22-48 h as reported | D3 | 3.2 If data were missing, was bias from missingness unlikely? | Not applicable | Third-party balanced block randomization; 6-14 d washout; mixed model adjusted for period. | Low |
| Dempsey 2017 | Primary CGM result; 22-48 h as reported | D4 | 4.1 Was the CGM/FGM measurement method appropriate? | Yes | Third-party balanced block randomization; 6-14 d washout; mixed model adjusted for period. | Low |
| Dempsey 2017 | Primary CGM result; 22-48 h as reported | D4 | 4.2 Could measurement or ascertainment differ between conditions? | No | Third-party balanced block randomization; 6-14 d washout; mixed model adjusted for period. | Low |
| Dempsey 2017 | Primary CGM result; 22-48 h as reported | D4 | 4.3 Could awareness of condition influence the recorded outcome? | No | Third-party balanced block randomization; 6-14 d washout; mixed model adjusted for period. | Low |
| Dempsey 2017 | Primary CGM result; 22-48 h as reported | D5 | 5.1 Was the reported analysis selected according to a prespecified plan? | Yes | Third-party balanced block randomization; 6-14 d washout; mixed model adjusted for period. | Low |
| Dempsey 2017 | Primary CGM result; 22-48 h as reported | D5 | 5.2 Was selection from multiple eligible outcome measurements unlikely? | Probably yes | Third-party balanced block randomization; 6-14 d washout; mixed model adjusted for period. | Low |
| Dempsey 2017 | Primary CGM result; 22-48 h as reported | D5 | 5.3 Was selection from multiple eligible analyses unlikely? | Probably yes | Third-party balanced block randomization; 6-14 d washout; mixed model adjusted for period. | Low |
| Duvivier 2017 | Primary CGM result; 22-48 h as reported | D1 | 1.1 Was the allocation sequence random? | Yes | Blocked randomization with sealed opaque envelopes; >=10 d washout; objective CGM. | Low |
| Duvivier 2017 | Primary CGM result; 22-48 h as reported | D1 | 1.2 Was the allocation sequence concealed until enrolment and assignment? | Yes | Blocked randomization with sealed opaque envelopes; >=10 d washout; objective CGM. | Low |
| Duvivier 2017 | Primary CGM result; 22-48 h as reported | D1 | 1.3 Did baseline or period-start differences suggest a problem with randomization? | Probably no | Blocked randomization with sealed opaque envelopes; >=10 d washout; objective CGM. | Low |
| Duvivier 2017 | Primary CGM result; 22-48 h as reported | Period/carryover | S1 Was the crossover design suitable for this acute outcome? | Yes | Blocked randomization with sealed opaque envelopes; >=10 d washout; objective CGM. | Low |
| Duvivier 2017 | Primary CGM result; 22-48 h as reported | Period/carryover | S2 Was the washout sufficient to make important carryover unlikely? | Yes | Blocked randomization with sealed opaque envelopes; >=10 d washout; objective CGM. | Low |
| Duvivier 2017 | Primary CGM result; 22-48 h as reported | Period/carryover | S3 Were period/order effects controlled or shown to be negligible? | Probably yes | Blocked randomization with sealed opaque envelopes; >=10 d washout; objective CGM. | Low |
| Duvivier 2017 | Primary CGM result; 22-48 h as reported | D2 | 2.1 Were participants aware of their assigned intervention? | Yes | Blocked randomization with sealed opaque envelopes; >=10 d washout; objective CGM. | Low |
| Duvivier 2017 | Primary CGM result; 22-48 h as reported | D2 | 2.2 Were intervention providers aware of assigned intervention? | Yes | Blocked randomization with sealed opaque envelopes; >=10 d washout; objective CGM. | Low |
| Duvivier 2017 | Primary CGM result; 22-48 h as reported | D2 | 2.3 Were there trial-context deviations beyond the intended protocol? | No | Blocked randomization with sealed opaque envelopes; >=10 d washout; objective CGM. | Low |
| Duvivier 2017 | Primary CGM result; 22-48 h as reported | D2 | 2.4 Were any deviations unbalanced and likely to affect the result? | Not applicable | Blocked randomization with sealed opaque envelopes; >=10 d washout; objective CGM. | Low |
| Duvivier 2017 | Primary CGM result; 22-48 h as reported | D2 | 2.5 Was an appropriate assigned-intervention analysis used? | Yes | Blocked randomization with sealed opaque envelopes; >=10 d washout; objective CGM. | Low |
| Duvivier 2017 | Primary CGM result; 22-48 h as reported | D3 | 3.1 Were outcome data available for all or nearly all randomized participants/periods? | Probably yes | Blocked randomization with sealed opaque envelopes; >=10 d washout; objective CGM. | Low |
| Duvivier 2017 | Primary CGM result; 22-48 h as reported | D3 | 3.2 If data were missing, was bias from missingness unlikely? | Not applicable | Blocked randomization with sealed opaque envelopes; >=10 d washout; objective CGM. | Low |
| Duvivier 2017 | Primary CGM result; 22-48 h as reported | D4 | 4.1 Was the CGM/FGM measurement method appropriate? | Yes | Blocked randomization with sealed opaque envelopes; >=10 d washout; objective CGM. | Low |
| Duvivier 2017 | Primary CGM result; 22-48 h as reported | D4 | 4.2 Could measurement or ascertainment differ between conditions? | No | Blocked randomization with sealed opaque envelopes; >=10 d washout; objective CGM. | Low |
| Duvivier 2017 | Primary CGM result; 22-48 h as reported | D4 | 4.3 Could awareness of condition influence the recorded outcome? | No | Blocked randomization with sealed opaque envelopes; >=10 d washout; objective CGM. | Low |
| Duvivier 2017 | Primary CGM result; 22-48 h as reported | D5 | 5.1 Was the reported analysis selected according to a prespecified plan? | Yes | Blocked randomization with sealed opaque envelopes; >=10 d washout; objective CGM. | Low |
| Duvivier 2017 | Primary CGM result; 22-48 h as reported | D5 | 5.2 Was selection from multiple eligible outcome measurements unlikely? | Probably yes | Blocked randomization with sealed opaque envelopes; >=10 d washout; objective CGM. | Low |
| Duvivier 2017 | Primary CGM result; 22-48 h as reported | D5 | 5.3 Was selection from multiple eligible analyses unlikely? | Probably yes | Blocked randomization with sealed opaque envelopes; >=10 d washout; objective CGM. | Low |
| Haxhi 2016 | Primary CGM result; 22-48 h as reported | D1 | 1.1 Was the allocation sequence random? | Probably yes | Randomized crossover with >=1 week separation; concealment and prespecified analysis information were not reported. | Some concerns |
| Haxhi 2016 | Primary CGM result; 22-48 h as reported | D1 | 1.2 Was the allocation sequence concealed until enrolment and assignment? | No information | Randomized crossover with >=1 week separation; concealment and prespecified analysis information were not reported. | Some concerns |
| Haxhi 2016 | Primary CGM result; 22-48 h as reported | D1 | 1.3 Did baseline or period-start differences suggest a problem with randomization? | Probably no | Randomized crossover with >=1 week separation; concealment and prespecified analysis information were not reported. | Some concerns |
| Haxhi 2016 | Primary CGM result; 22-48 h as reported | Period/carryover | S1 Was the crossover design suitable for this acute outcome? | Yes | Randomized crossover with >=1 week separation; concealment and prespecified analysis information were not reported. | Some concerns |
| Haxhi 2016 | Primary CGM result; 22-48 h as reported | Period/carryover | S2 Was the washout sufficient to make important carryover unlikely? | Probably yes | Randomized crossover with >=1 week separation; concealment and prespecified analysis information were not reported. | Some concerns |
| Haxhi 2016 | Primary CGM result; 22-48 h as reported | Period/carryover | S3 Were period/order effects controlled or shown to be negligible? | No information | Randomized crossover with >=1 week separation; concealment and prespecified analysis information were not reported. | Some concerns |
| Haxhi 2016 | Primary CGM result; 22-48 h as reported | D2 | 2.1 Were participants aware of their assigned intervention? | Yes | Randomized crossover with >=1 week separation; concealment and prespecified analysis information were not reported. | Some concerns |
| Haxhi 2016 | Primary CGM result; 22-48 h as reported | D2 | 2.2 Were intervention providers aware of assigned intervention? | Yes | Randomized crossover with >=1 week separation; concealment and prespecified analysis information were not reported. | Some concerns |
| Haxhi 2016 | Primary CGM result; 22-48 h as reported | D2 | 2.3 Were there trial-context deviations beyond the intended protocol? | No | Randomized crossover with >=1 week separation; concealment and prespecified analysis information were not reported. | Some concerns |
| Haxhi 2016 | Primary CGM result; 22-48 h as reported | D2 | 2.4 Were any deviations unbalanced and likely to affect the result? | Not applicable | Randomized crossover with >=1 week separation; concealment and prespecified analysis information were not reported. | Some concerns |
| Haxhi 2016 | Primary CGM result; 22-48 h as reported | D2 | 2.5 Was an appropriate assigned-intervention analysis used? | Yes | Randomized crossover with >=1 week separation; concealment and prespecified analysis information were not reported. | Some concerns |
| Haxhi 2016 | Primary CGM result; 22-48 h as reported | D3 | 3.1 Were outcome data available for all or nearly all randomized participants/periods? | Probably yes | Randomized crossover with >=1 week separation; concealment and prespecified analysis information were not reported. | Some concerns |
| Haxhi 2016 | Primary CGM result; 22-48 h as reported | D3 | 3.2 If data were missing, was bias from missingness unlikely? | Not applicable | Randomized crossover with >=1 week separation; concealment and prespecified analysis information were not reported. | Some concerns |
| Haxhi 2016 | Primary CGM result; 22-48 h as reported | D4 | 4.1 Was the CGM/FGM measurement method appropriate? | Yes | Randomized crossover with >=1 week separation; concealment and prespecified analysis information were not reported. | Some concerns |
| Haxhi 2016 | Primary CGM result; 22-48 h as reported | D4 | 4.2 Could measurement or ascertainment differ between conditions? | No | Randomized crossover with >=1 week separation; concealment and prespecified analysis information were not reported. | Some concerns |
| Haxhi 2016 | Primary CGM result; 22-48 h as reported | D4 | 4.3 Could awareness of condition influence the recorded outcome? | No | Randomized crossover with >=1 week separation; concealment and prespecified analysis information were not reported. | Some concerns |
| Haxhi 2016 | Primary CGM result; 22-48 h as reported | D5 | 5.1 Was the reported analysis selected according to a prespecified plan? | Probably no | Randomized crossover with >=1 week separation; concealment and prespecified analysis information were not reported. | Some concerns |
| Haxhi 2016 | Primary CGM result; 22-48 h as reported | D5 | 5.2 Was selection from multiple eligible outcome measurements unlikely? | No information | Randomized crossover with >=1 week separation; concealment and prespecified analysis information were not reported. | Some concerns |
| Haxhi 2016 | Primary CGM result; 22-48 h as reported | D5 | 5.3 Was selection from multiple eligible analyses unlikely? | No information | Randomized crossover with >=1 week separation; concealment and prespecified analysis information were not reported. | Some concerns |
| Homer 2021 | Overall mean glucose, 22 h | D1 | 1.1 Was the allocation sequence random? | Yes | Third-party balanced block randomization; 6-14 d washout; 24/24 completed all three conditions. Nine missing pre-breakfast baseline values were handled only for meal-specific iAUC and did not make the 22-h summary result incomplete. | Low |
| Homer 2021 | Overall mean glucose, 22 h | D1 | 1.2 Was the allocation sequence concealed until enrolment and assignment? | Yes | Third-party balanced block randomization; 6-14 d washout; 24/24 completed all three conditions. Nine missing pre-breakfast baseline values were handled only for meal-specific iAUC and did not make the 22-h summary result incomplete. | Low |
| Homer 2021 | Overall mean glucose, 22 h | D1 | 1.3 Did baseline or period-start differences suggest a problem with randomization? | Probably no | Third-party balanced block randomization; 6-14 d washout; 24/24 completed all three conditions. Nine missing pre-breakfast baseline values were handled only for meal-specific iAUC and did not make the 22-h summary result incomplete. | Low |
| Homer 2021 | Overall mean glucose, 22 h | Period/carryover | S1 Was the crossover design suitable for this acute outcome? | Yes | Third-party balanced block randomization; 6-14 d washout; 24/24 completed all three conditions. Nine missing pre-breakfast baseline values were handled only for meal-specific iAUC and did not make the 22-h summary result incomplete. | Low |
| Homer 2021 | Overall mean glucose, 22 h | Period/carryover | S2 Was the washout sufficient to make important carryover unlikely? | Yes | Third-party balanced block randomization; 6-14 d washout; 24/24 completed all three conditions. Nine missing pre-breakfast baseline values were handled only for meal-specific iAUC and did not make the 22-h summary result incomplete. | Low |
| Homer 2021 | Overall mean glucose, 22 h | Period/carryover | S3 Were period/order effects controlled or shown to be negligible? | Yes | Third-party balanced block randomization; 6-14 d washout; 24/24 completed all three conditions. Nine missing pre-breakfast baseline values were handled only for meal-specific iAUC and did not make the 22-h summary result incomplete. | Low |
| Homer 2021 | Overall mean glucose, 22 h | D2 | 2.1 Were participants aware of their assigned intervention? | Yes | Third-party balanced block randomization; 6-14 d washout; 24/24 completed all three conditions. Nine missing pre-breakfast baseline values were handled only for meal-specific iAUC and did not make the 22-h summary result incomplete. | Low |
| Homer 2021 | Overall mean glucose, 22 h | D2 | 2.2 Were intervention providers aware of assigned intervention? | Yes | Third-party balanced block randomization; 6-14 d washout; 24/24 completed all three conditions. Nine missing pre-breakfast baseline values were handled only for meal-specific iAUC and did not make the 22-h summary result incomplete. | Low |
| Homer 2021 | Overall mean glucose, 22 h | D2 | 2.3 Were there trial-context deviations beyond the intended protocol? | No | Third-party balanced block randomization; 6-14 d washout; 24/24 completed all three conditions. Nine missing pre-breakfast baseline values were handled only for meal-specific iAUC and did not make the 22-h summary result incomplete. | Low |
| Homer 2021 | Overall mean glucose, 22 h | D2 | 2.4 Were any deviations unbalanced and likely to affect the result? | Not applicable | Third-party balanced block randomization; 6-14 d washout; 24/24 completed all three conditions. Nine missing pre-breakfast baseline values were handled only for meal-specific iAUC and did not make the 22-h summary result incomplete. | Low |
| Homer 2021 | Overall mean glucose, 22 h | D2 | 2.5 Was an appropriate assigned-intervention analysis used? | Yes | Third-party balanced block randomization; 6-14 d washout; 24/24 completed all three conditions. Nine missing pre-breakfast baseline values were handled only for meal-specific iAUC and did not make the 22-h summary result incomplete. | Low |
| Homer 2021 | Overall mean glucose, 22 h | D3 | 3.1 Were outcome data available for all or nearly all randomized participants/periods? | Probably yes | Third-party balanced block randomization; 6-14 d washout; 24/24 completed all three conditions. Nine missing pre-breakfast baseline values were handled only for meal-specific iAUC and did not make the 22-h summary result incomplete. | Low |
| Homer 2021 | Overall mean glucose, 22 h | D3 | 3.2 If data were missing, was bias from missingness unlikely? | Not applicable | Third-party balanced block randomization; 6-14 d washout; 24/24 completed all three conditions. Nine missing pre-breakfast baseline values were handled only for meal-specific iAUC and did not make the 22-h summary result incomplete. | Low |
| Homer 2021 | Overall mean glucose, 22 h | D4 | 4.1 Was the CGM/FGM measurement method appropriate? | Yes | Third-party balanced block randomization; 6-14 d washout; 24/24 completed all three conditions. Nine missing pre-breakfast baseline values were handled only for meal-specific iAUC and did not make the 22-h summary result incomplete. | Low |
| Homer 2021 | Overall mean glucose, 22 h | D4 | 4.2 Could measurement or ascertainment differ between conditions? | No | Third-party balanced block randomization; 6-14 d washout; 24/24 completed all three conditions. Nine missing pre-breakfast baseline values were handled only for meal-specific iAUC and did not make the 22-h summary result incomplete. | Low |
| Homer 2021 | Overall mean glucose, 22 h | D4 | 4.3 Could awareness of condition influence the recorded outcome? | No | Third-party balanced block randomization; 6-14 d washout; 24/24 completed all three conditions. Nine missing pre-breakfast baseline values were handled only for meal-specific iAUC and did not make the 22-h summary result incomplete. | Low |
| Homer 2021 | Overall mean glucose, 22 h | D5 | 5.1 Was the reported analysis selected according to a prespecified plan? | Yes | Third-party balanced block randomization; 6-14 d washout; 24/24 completed all three conditions. Nine missing pre-breakfast baseline values were handled only for meal-specific iAUC and did not make the 22-h summary result incomplete. | Low |
| Homer 2021 | Overall mean glucose, 22 h | D5 | 5.2 Was selection from multiple eligible outcome measurements unlikely? | Probably yes | Third-party balanced block randomization; 6-14 d washout; 24/24 completed all three conditions. Nine missing pre-breakfast baseline values were handled only for meal-specific iAUC and did not make the 22-h summary result incomplete. | Low |
| Homer 2021 | Overall mean glucose, 22 h | D5 | 5.3 Was selection from multiple eligible analyses unlikely? | Probably yes | Third-party balanced block randomization; 6-14 d washout; 24/24 completed all three conditions. Nine missing pre-breakfast baseline values were handled only for meal-specific iAUC and did not make the 22-h summary result incomplete. | Low |
| Metcalfe 2018 | Overall mean glucose, 24 h | D1 | 1.1 Was the allocation sequence random? | Yes | Registered randomized four-condition acute crossover (NCT03082859); condition order assigned by envelope method; >=5 d washout; 11/11 completed all conditions; objective CGM. | Low |
| Metcalfe 2018 | Overall mean glucose, 24 h | D1 | 1.2 Was the allocation sequence concealed until enrolment and assignment? | Probably yes | Registered randomized four-condition acute crossover (NCT03082859); condition order assigned by envelope method; >=5 d washout; 11/11 completed all conditions; objective CGM. | Low |
| Metcalfe 2018 | Overall mean glucose, 24 h | D1 | 1.3 Did baseline or period-start differences suggest a problem with randomization? | Probably no | Registered randomized four-condition acute crossover (NCT03082859); condition order assigned by envelope method; >=5 d washout; 11/11 completed all conditions; objective CGM. | Low |
| Metcalfe 2018 | Overall mean glucose, 24 h | Period/carryover | S1 Was the crossover design suitable for this acute outcome? | Yes | Registered randomized four-condition acute crossover (NCT03082859); condition order assigned by envelope method; >=5 d washout; 11/11 completed all conditions; objective CGM. | Low |
| Metcalfe 2018 | Overall mean glucose, 24 h | Period/carryover | S2 Was the washout sufficient to make important carryover unlikely? | Yes | Registered randomized four-condition acute crossover (NCT03082859); condition order assigned by envelope method; >=5 d washout; 11/11 completed all conditions; objective CGM. | Low |
| Metcalfe 2018 | Overall mean glucose, 24 h | Period/carryover | S3 Were period/order effects controlled or shown to be negligible? | Probably yes | Registered randomized four-condition acute crossover (NCT03082859); condition order assigned by envelope method; >=5 d washout; 11/11 completed all conditions; objective CGM. | Low |
| Metcalfe 2018 | Overall mean glucose, 24 h | D2 | 2.1 Were participants aware of their assigned intervention? | Yes | Registered randomized four-condition acute crossover (NCT03082859); condition order assigned by envelope method; >=5 d washout; 11/11 completed all conditions; objective CGM. | Low |
| Metcalfe 2018 | Overall mean glucose, 24 h | D2 | 2.2 Were intervention providers aware of assigned intervention? | Yes | Registered randomized four-condition acute crossover (NCT03082859); condition order assigned by envelope method; >=5 d washout; 11/11 completed all conditions; objective CGM. | Low |
| Metcalfe 2018 | Overall mean glucose, 24 h | D2 | 2.3 Were there trial-context deviations beyond the intended protocol? | No | Registered randomized four-condition acute crossover (NCT03082859); condition order assigned by envelope method; >=5 d washout; 11/11 completed all conditions; objective CGM. | Low |
| Metcalfe 2018 | Overall mean glucose, 24 h | D2 | 2.4 Were any deviations unbalanced and likely to affect the result? | Not applicable | Registered randomized four-condition acute crossover (NCT03082859); condition order assigned by envelope method; >=5 d washout; 11/11 completed all conditions; objective CGM. | Low |
| Metcalfe 2018 | Overall mean glucose, 24 h | D2 | 2.5 Was an appropriate assigned-intervention analysis used? | Yes | Registered randomized four-condition acute crossover (NCT03082859); condition order assigned by envelope method; >=5 d washout; 11/11 completed all conditions; objective CGM. | Low |
| Metcalfe 2018 | Overall mean glucose, 24 h | D3 | 3.1 Were outcome data available for all or nearly all randomized participants/periods? | Probably yes | Registered randomized four-condition acute crossover (NCT03082859); condition order assigned by envelope method; >=5 d washout; 11/11 completed all conditions; objective CGM. | Low |
| Metcalfe 2018 | Overall mean glucose, 24 h | D3 | 3.2 If data were missing, was bias from missingness unlikely? | Not applicable | Registered randomized four-condition acute crossover (NCT03082859); condition order assigned by envelope method; >=5 d washout; 11/11 completed all conditions; objective CGM. | Low |
| Metcalfe 2018 | Overall mean glucose, 24 h | D4 | 4.1 Was the CGM/FGM measurement method appropriate? | Yes | Registered randomized four-condition acute crossover (NCT03082859); condition order assigned by envelope method; >=5 d washout; 11/11 completed all conditions; objective CGM. | Low |
| Metcalfe 2018 | Overall mean glucose, 24 h | D4 | 4.2 Could measurement or ascertainment differ between conditions? | No | Registered randomized four-condition acute crossover (NCT03082859); condition order assigned by envelope method; >=5 d washout; 11/11 completed all conditions; objective CGM. | Low |
| Metcalfe 2018 | Overall mean glucose, 24 h | D4 | 4.3 Could awareness of condition influence the recorded outcome? | No | Registered randomized four-condition acute crossover (NCT03082859); condition order assigned by envelope method; >=5 d washout; 11/11 completed all conditions; objective CGM. | Low |
| Metcalfe 2018 | Overall mean glucose, 24 h | D5 | 5.1 Was the reported analysis selected according to a prespecified plan? | Yes | Registered randomized four-condition acute crossover (NCT03082859); condition order assigned by envelope method; >=5 d washout; 11/11 completed all conditions; objective CGM. | Low |
| Metcalfe 2018 | Overall mean glucose, 24 h | D5 | 5.2 Was selection from multiple eligible outcome measurements unlikely? | Probably yes | Registered randomized four-condition acute crossover (NCT03082859); condition order assigned by envelope method; >=5 d washout; 11/11 completed all conditions; objective CGM. | Low |
| Metcalfe 2018 | Overall mean glucose, 24 h | D5 | 5.3 Was selection from multiple eligible analyses unlikely? | Probably yes | Registered randomized four-condition acute crossover (NCT03082859); condition order assigned by envelope method; >=5 d washout; 11/11 completed all conditions; objective CGM. | Low |
| van Dijk 2013 | Primary CGM result; 22-48 h as reported | D1 | 1.1 Was the allocation sequence random? | Probably yes | Randomized crossover with >=1 week separation; sequence and carryover analysis details were insufficient. | Some concerns |
| van Dijk 2013 | Primary CGM result; 22-48 h as reported | D1 | 1.2 Was the allocation sequence concealed until enrolment and assignment? | No information | Randomized crossover with >=1 week separation; sequence and carryover analysis details were insufficient. | Some concerns |
| van Dijk 2013 | Primary CGM result; 22-48 h as reported | D1 | 1.3 Did baseline or period-start differences suggest a problem with randomization? | Probably no | Randomized crossover with >=1 week separation; sequence and carryover analysis details were insufficient. | Some concerns |
| van Dijk 2013 | Primary CGM result; 22-48 h as reported | Period/carryover | S1 Was the crossover design suitable for this acute outcome? | Yes | Randomized crossover with >=1 week separation; sequence and carryover analysis details were insufficient. | Some concerns |
| van Dijk 2013 | Primary CGM result; 22-48 h as reported | Period/carryover | S2 Was the washout sufficient to make important carryover unlikely? | Probably yes | Randomized crossover with >=1 week separation; sequence and carryover analysis details were insufficient. | Some concerns |
| van Dijk 2013 | Primary CGM result; 22-48 h as reported | Period/carryover | S3 Were period/order effects controlled or shown to be negligible? | No information | Randomized crossover with >=1 week separation; sequence and carryover analysis details were insufficient. | Some concerns |
| van Dijk 2013 | Primary CGM result; 22-48 h as reported | D2 | 2.1 Were participants aware of their assigned intervention? | Yes | Randomized crossover with >=1 week separation; sequence and carryover analysis details were insufficient. | Some concerns |
| van Dijk 2013 | Primary CGM result; 22-48 h as reported | D2 | 2.2 Were intervention providers aware of assigned intervention? | Yes | Randomized crossover with >=1 week separation; sequence and carryover analysis details were insufficient. | Some concerns |
| van Dijk 2013 | Primary CGM result; 22-48 h as reported | D2 | 2.3 Were there trial-context deviations beyond the intended protocol? | No | Randomized crossover with >=1 week separation; sequence and carryover analysis details were insufficient. | Some concerns |
| van Dijk 2013 | Primary CGM result; 22-48 h as reported | D2 | 2.4 Were any deviations unbalanced and likely to affect the result? | Not applicable | Randomized crossover with >=1 week separation; sequence and carryover analysis details were insufficient. | Some concerns |
| van Dijk 2013 | Primary CGM result; 22-48 h as reported | D2 | 2.5 Was an appropriate assigned-intervention analysis used? | Yes | Randomized crossover with >=1 week separation; sequence and carryover analysis details were insufficient. | Some concerns |
| van Dijk 2013 | Primary CGM result; 22-48 h as reported | D3 | 3.1 Were outcome data available for all or nearly all randomized participants/periods? | Probably yes | Randomized crossover with >=1 week separation; sequence and carryover analysis details were insufficient. | Some concerns |
| van Dijk 2013 | Primary CGM result; 22-48 h as reported | D3 | 3.2 If data were missing, was bias from missingness unlikely? | Not applicable | Randomized crossover with >=1 week separation; sequence and carryover analysis details were insufficient. | Some concerns |
| van Dijk 2013 | Primary CGM result; 22-48 h as reported | D4 | 4.1 Was the CGM/FGM measurement method appropriate? | Yes | Randomized crossover with >=1 week separation; sequence and carryover analysis details were insufficient. | Some concerns |
| van Dijk 2013 | Primary CGM result; 22-48 h as reported | D4 | 4.2 Could measurement or ascertainment differ between conditions? | No | Randomized crossover with >=1 week separation; sequence and carryover analysis details were insufficient. | Some concerns |
| van Dijk 2013 | Primary CGM result; 22-48 h as reported | D4 | 4.3 Could awareness of condition influence the recorded outcome? | No | Randomized crossover with >=1 week separation; sequence and carryover analysis details were insufficient. | Some concerns |
| van Dijk 2013 | Primary CGM result; 22-48 h as reported | D5 | 5.1 Was the reported analysis selected according to a prespecified plan? | Yes | Randomized crossover with >=1 week separation; sequence and carryover analysis details were insufficient. | Some concerns |
| van Dijk 2013 | Primary CGM result; 22-48 h as reported | D5 | 5.2 Was selection from multiple eligible outcome measurements unlikely? | Probably yes | Randomized crossover with >=1 week separation; sequence and carryover analysis details were insufficient. | Some concerns |
| van Dijk 2013 | Primary CGM result; 22-48 h as reported | D5 | 5.3 Was selection from multiple eligible analyses unlikely? | Probably yes | Randomized crossover with >=1 week separation; sequence and carryover analysis details were insufficient. | Some concerns |
| Gillen 2012 | Primary CGM result; study-specific window | D1 | 1.1 Was the allocation sequence random? | No | Controlled within-person study, but CTL always preceded HIT; objective CGM and complete data for seven participants. | High |
| Gillen 2012 | Primary CGM result; study-specific window | D1 | 1.2 Was the allocation sequence concealed until enrolment and assignment? | No | Controlled within-person study, but CTL always preceded HIT; objective CGM and complete data for seven participants. | High |
| Gillen 2012 | Primary CGM result; study-specific window | D1 | 1.3 Did baseline or period-start differences suggest a problem with randomization? | Probably yes | Controlled within-person study, but CTL always preceded HIT; objective CGM and complete data for seven participants. | High |
| Gillen 2012 | Primary CGM result; study-specific window | Period/carryover | S1 Was the crossover design suitable for this acute outcome? | Yes | Controlled within-person study, but CTL always preceded HIT; objective CGM and complete data for seven participants. | High |
| Gillen 2012 | Primary CGM result; study-specific window | Period/carryover | S2 Was the washout sufficient to make important carryover unlikely? | Probably yes | Controlled within-person study, but CTL always preceded HIT; objective CGM and complete data for seven participants. | High |
| Gillen 2012 | Primary CGM result; study-specific window | Period/carryover | S3 Were period/order effects controlled or shown to be negligible? | No | Controlled within-person study, but CTL always preceded HIT; objective CGM and complete data for seven participants. | High |
| Gillen 2012 | Primary CGM result; study-specific window | D2 | 2.1 Were participants aware of their assigned intervention? | Yes | Controlled within-person study, but CTL always preceded HIT; objective CGM and complete data for seven participants. | Low |
| Gillen 2012 | Primary CGM result; study-specific window | D2 | 2.2 Were intervention providers aware of assigned intervention? | Yes | Controlled within-person study, but CTL always preceded HIT; objective CGM and complete data for seven participants. | Low |
| Gillen 2012 | Primary CGM result; study-specific window | D2 | 2.3 Were there trial-context deviations beyond the intended protocol? | No | Controlled within-person study, but CTL always preceded HIT; objective CGM and complete data for seven participants. | Low |
| Gillen 2012 | Primary CGM result; study-specific window | D2 | 2.4 Were any deviations unbalanced and likely to affect the result? | Not applicable | Controlled within-person study, but CTL always preceded HIT; objective CGM and complete data for seven participants. | Low |
| Gillen 2012 | Primary CGM result; study-specific window | D2 | 2.5 Was an appropriate assigned-intervention analysis used? | Yes | Controlled within-person study, but CTL always preceded HIT; objective CGM and complete data for seven participants. | Low |
| Gillen 2012 | Primary CGM result; study-specific window | D3 | 3.1 Were outcome data available for all or nearly all randomized participants/periods? | Yes | Controlled within-person study, but CTL always preceded HIT; objective CGM and complete data for seven participants. | Low |
| Gillen 2012 | Primary CGM result; study-specific window | D3 | 3.2 If data were missing, was bias from missingness unlikely? | Not applicable | Controlled within-person study, but CTL always preceded HIT; objective CGM and complete data for seven participants. | Low |
| Gillen 2012 | Primary CGM result; study-specific window | D4 | 4.1 Was the CGM/FGM measurement method appropriate? | Yes | Controlled within-person study, but CTL always preceded HIT; objective CGM and complete data for seven participants. | Low |
| Gillen 2012 | Primary CGM result; study-specific window | D4 | 4.2 Could measurement or ascertainment differ between conditions? | No | Controlled within-person study, but CTL always preceded HIT; objective CGM and complete data for seven participants. | Low |
| Gillen 2012 | Primary CGM result; study-specific window | D4 | 4.3 Could awareness of condition influence the recorded outcome? | No | Controlled within-person study, but CTL always preceded HIT; objective CGM and complete data for seven participants. | Low |
| Gillen 2012 | Primary CGM result; study-specific window | D5 | 5.1 Was the reported analysis selected according to a prespecified plan? | No information | Controlled within-person study, but CTL always preceded HIT; objective CGM and complete data for seven participants. | Some concerns |
| Gillen 2012 | Primary CGM result; study-specific window | D5 | 5.2 Was selection from multiple eligible outcome measurements unlikely? | Probably no | Controlled within-person study, but CTL always preceded HIT; objective CGM and complete data for seven participants. | Some concerns |
| Gillen 2012 | Primary CGM result; study-specific window | D5 | 5.3 Was selection from multiple eligible analyses unlikely? | No information | Controlled within-person study, but CTL always preceded HIT; objective CGM and complete data for seven participants. | Some concerns |
| Myette-Côté 2018 | Primary CGM result; study-specific window | D1 | 1.1 Was the allocation sequence random? | Yes | Computer-randomized crossover with 9-14-day washout; 11/16 completed; objective CGM; pairwise Fisher-LSD results reported. | Low |
| Myette-Côté 2018 | Primary CGM result; study-specific window | D1 | 1.2 Was the allocation sequence concealed until enrolment and assignment? | Probably yes | Computer-randomized crossover with 9-14-day washout; 11/16 completed; objective CGM; pairwise Fisher-LSD results reported. | Low |
| Myette-Côté 2018 | Primary CGM result; study-specific window | D1 | 1.3 Did baseline or period-start differences suggest a problem with randomization? | Probably no | Computer-randomized crossover with 9-14-day washout; 11/16 completed; objective CGM; pairwise Fisher-LSD results reported. | Low |
| Myette-Côté 2018 | Primary CGM result; study-specific window | Period/carryover | S1 Was the crossover design suitable for this acute outcome? | Yes | Computer-randomized crossover with 9-14-day washout; 11/16 completed; objective CGM; pairwise Fisher-LSD results reported. | Low |
| Myette-Côté 2018 | Primary CGM result; study-specific window | Period/carryover | S2 Was the washout sufficient to make important carryover unlikely? | Yes | Computer-randomized crossover with 9-14-day washout; 11/16 completed; objective CGM; pairwise Fisher-LSD results reported. | Low |
| Myette-Côté 2018 | Primary CGM result; study-specific window | Period/carryover | S3 Were period/order effects controlled or shown to be negligible? | Yes | Computer-randomized crossover with 9-14-day washout; 11/16 completed; objective CGM; pairwise Fisher-LSD results reported. | Low |
| Myette-Côté 2018 | Primary CGM result; study-specific window | D2 | 2.1 Were participants aware of their assigned intervention? | Yes | Computer-randomized crossover with 9-14-day washout; 11/16 completed; objective CGM; pairwise Fisher-LSD results reported. | Low |
| Myette-Côté 2018 | Primary CGM result; study-specific window | D2 | 2.2 Were intervention providers aware of assigned intervention? | Yes | Computer-randomized crossover with 9-14-day washout; 11/16 completed; objective CGM; pairwise Fisher-LSD results reported. | Low |
| Myette-Côté 2018 | Primary CGM result; study-specific window | D2 | 2.3 Were there trial-context deviations beyond the intended protocol? | No | Computer-randomized crossover with 9-14-day washout; 11/16 completed; objective CGM; pairwise Fisher-LSD results reported. | Low |
| Myette-Côté 2018 | Primary CGM result; study-specific window | D2 | 2.4 Were any deviations unbalanced and likely to affect the result? | Not applicable | Computer-randomized crossover with 9-14-day washout; 11/16 completed; objective CGM; pairwise Fisher-LSD results reported. | Low |
| Myette-Côté 2018 | Primary CGM result; study-specific window | D2 | 2.5 Was an appropriate assigned-intervention analysis used? | Yes | Computer-randomized crossover with 9-14-day washout; 11/16 completed; objective CGM; pairwise Fisher-LSD results reported. | Low |
| Myette-Côté 2018 | Primary CGM result; study-specific window | D3 | 3.1 Were outcome data available for all or nearly all randomized participants/periods? | No | Computer-randomized crossover with 9-14-day washout; 11/16 completed; objective CGM; pairwise Fisher-LSD results reported. | Some concerns |
| Myette-Côté 2018 | Primary CGM result; study-specific window | D3 | 3.2 If data were missing, was bias from missingness unlikely? | Probably no | Computer-randomized crossover with 9-14-day washout; 11/16 completed; objective CGM; pairwise Fisher-LSD results reported. | Some concerns |
| Myette-Côté 2018 | Primary CGM result; study-specific window | D4 | 4.1 Was the CGM/FGM measurement method appropriate? | Yes | Computer-randomized crossover with 9-14-day washout; 11/16 completed; objective CGM; pairwise Fisher-LSD results reported. | Low |
| Myette-Côté 2018 | Primary CGM result; study-specific window | D4 | 4.2 Could measurement or ascertainment differ between conditions? | No | Computer-randomized crossover with 9-14-day washout; 11/16 completed; objective CGM; pairwise Fisher-LSD results reported. | Low |
| Myette-Côté 2018 | Primary CGM result; study-specific window | D4 | 4.3 Could awareness of condition influence the recorded outcome? | No | Computer-randomized crossover with 9-14-day washout; 11/16 completed; objective CGM; pairwise Fisher-LSD results reported. | Low |
| Myette-Côté 2018 | Primary CGM result; study-specific window | D5 | 5.1 Was the reported analysis selected according to a prespecified plan? | No information | Computer-randomized crossover with 9-14-day washout; 11/16 completed; objective CGM; pairwise Fisher-LSD results reported. | Some concerns |
| Myette-Côté 2018 | Primary CGM result; study-specific window | D5 | 5.2 Was selection from multiple eligible outcome measurements unlikely? | Probably no | Computer-randomized crossover with 9-14-day washout; 11/16 completed; objective CGM; pairwise Fisher-LSD results reported. | Some concerns |
| Myette-Côté 2018 | Primary CGM result; study-specific window | D5 | 5.3 Was selection from multiple eligible analyses unlikely? | Probably no | Computer-randomized crossover with 9-14-day washout; 11/16 completed; objective CGM; pairwise Fisher-LSD results reported. | Some concerns |
| Terada 2016 | Primary CGM result; study-specific window | D1 | 1.1 Was the allocation sequence random? | Yes | Randomized five-condition crossover with 48-hour washout; objective CGM; concealment and reasons for outcome-specific n were incompletely reported. | Some concerns |
| Terada 2016 | Primary CGM result; study-specific window | D1 | 1.2 Was the allocation sequence concealed until enrolment and assignment? | No information | Randomized five-condition crossover with 48-hour washout; objective CGM; concealment and reasons for outcome-specific n were incompletely reported. | Some concerns |
| Terada 2016 | Primary CGM result; study-specific window | D1 | 1.3 Did baseline or period-start differences suggest a problem with randomization? | Probably no | Randomized five-condition crossover with 48-hour washout; objective CGM; concealment and reasons for outcome-specific n were incompletely reported. | Some concerns |
| Terada 2016 | Primary CGM result; study-specific window | Period/carryover | S1 Was the crossover design suitable for this acute outcome? | Yes | Randomized five-condition crossover with 48-hour washout; objective CGM; concealment and reasons for outcome-specific n were incompletely reported. | Low |
| Terada 2016 | Primary CGM result; study-specific window | Period/carryover | S2 Was the washout sufficient to make important carryover unlikely? | Probably yes | Randomized five-condition crossover with 48-hour washout; objective CGM; concealment and reasons for outcome-specific n were incompletely reported. | Low |
| Terada 2016 | Primary CGM result; study-specific window | Period/carryover | S3 Were period/order effects controlled or shown to be negligible? | Probably yes | Randomized five-condition crossover with 48-hour washout; objective CGM; concealment and reasons for outcome-specific n were incompletely reported. | Low |
| Terada 2016 | Primary CGM result; study-specific window | D2 | 2.1 Were participants aware of their assigned intervention? | Yes | Randomized five-condition crossover with 48-hour washout; objective CGM; concealment and reasons for outcome-specific n were incompletely reported. | Low |
| Terada 2016 | Primary CGM result; study-specific window | D2 | 2.2 Were intervention providers aware of assigned intervention? | Yes | Randomized five-condition crossover with 48-hour washout; objective CGM; concealment and reasons for outcome-specific n were incompletely reported. | Low |
| Terada 2016 | Primary CGM result; study-specific window | D2 | 2.3 Were there trial-context deviations beyond the intended protocol? | No | Randomized five-condition crossover with 48-hour washout; objective CGM; concealment and reasons for outcome-specific n were incompletely reported. | Low |
| Terada 2016 | Primary CGM result; study-specific window | D2 | 2.4 Were any deviations unbalanced and likely to affect the result? | Not applicable | Randomized five-condition crossover with 48-hour washout; objective CGM; concealment and reasons for outcome-specific n were incompletely reported. | Low |
| Terada 2016 | Primary CGM result; study-specific window | D2 | 2.5 Was an appropriate assigned-intervention analysis used? | Yes | Randomized five-condition crossover with 48-hour washout; objective CGM; concealment and reasons for outcome-specific n were incompletely reported. | Low |
| Terada 2016 | Primary CGM result; study-specific window | D3 | 3.1 Were outcome data available for all or nearly all randomized participants/periods? | No | Randomized five-condition crossover with 48-hour washout; objective CGM; concealment and reasons for outcome-specific n were incompletely reported. | Some concerns |
| Terada 2016 | Primary CGM result; study-specific window | D3 | 3.2 If data were missing, was bias from missingness unlikely? | Probably no | Randomized five-condition crossover with 48-hour washout; objective CGM; concealment and reasons for outcome-specific n were incompletely reported. | Some concerns |
| Terada 2016 | Primary CGM result; study-specific window | D4 | 4.1 Was the CGM/FGM measurement method appropriate? | Yes | Randomized five-condition crossover with 48-hour washout; objective CGM; concealment and reasons for outcome-specific n were incompletely reported. | Low |
| Terada 2016 | Primary CGM result; study-specific window | D4 | 4.2 Could measurement or ascertainment differ between conditions? | No | Randomized five-condition crossover with 48-hour washout; objective CGM; concealment and reasons for outcome-specific n were incompletely reported. | Low |
| Terada 2016 | Primary CGM result; study-specific window | D4 | 4.3 Could awareness of condition influence the recorded outcome? | No | Randomized five-condition crossover with 48-hour washout; objective CGM; concealment and reasons for outcome-specific n were incompletely reported. | Low |
| Terada 2016 | Primary CGM result; study-specific window | D5 | 5.1 Was the reported analysis selected according to a prespecified plan? | No information | Randomized five-condition crossover with 48-hour washout; objective CGM; concealment and reasons for outcome-specific n were incompletely reported. | Some concerns |
| Terada 2016 | Primary CGM result; study-specific window | D5 | 5.2 Was selection from multiple eligible outcome measurements unlikely? | Probably no | Randomized five-condition crossover with 48-hour washout; objective CGM; concealment and reasons for outcome-specific n were incompletely reported. | Some concerns |
| Terada 2016 | Primary CGM result; study-specific window | D5 | 5.3 Was selection from multiple eligible analyses unlikely? | No information | Randomized five-condition crossover with 48-hour washout; objective CGM; concealment and reasons for outcome-specific n were incompletely reported. | Some concerns |
| de Hoogh 2025 | Primary CGM result; study-specific window | D1 | 1.1 Was the allocation sequence random? | Yes | Randomized repeated intervention periods with at least one-week washout and objective CGM; result-specific n and marginal estimands were incompletely reported. | Low |
| de Hoogh 2025 | Primary CGM result; study-specific window | D1 | 1.2 Was the allocation sequence concealed until enrolment and assignment? | Probably yes | Randomized repeated intervention periods with at least one-week washout and objective CGM; result-specific n and marginal estimands were incompletely reported. | Low |
| de Hoogh 2025 | Primary CGM result; study-specific window | D1 | 1.3 Did baseline or period-start differences suggest a problem with randomization? | Probably no | Randomized repeated intervention periods with at least one-week washout and objective CGM; result-specific n and marginal estimands were incompletely reported. | Low |
| de Hoogh 2025 | Primary CGM result; study-specific window | Period/carryover | S1 Was the crossover design suitable for this acute outcome? | Yes | Randomized repeated intervention periods with at least one-week washout and objective CGM; result-specific n and marginal estimands were incompletely reported. | Low |
| de Hoogh 2025 | Primary CGM result; study-specific window | Period/carryover | S2 Was the washout sufficient to make important carryover unlikely? | Yes | Randomized repeated intervention periods with at least one-week washout and objective CGM; result-specific n and marginal estimands were incompletely reported. | Low |
| de Hoogh 2025 | Primary CGM result; study-specific window | Period/carryover | S3 Were period/order effects controlled or shown to be negligible? | Yes | Randomized repeated intervention periods with at least one-week washout and objective CGM; result-specific n and marginal estimands were incompletely reported. | Low |
| de Hoogh 2025 | Primary CGM result; study-specific window | D2 | 2.1 Were participants aware of their assigned intervention? | Yes | Randomized repeated intervention periods with at least one-week washout and objective CGM; result-specific n and marginal estimands were incompletely reported. | Low |
| de Hoogh 2025 | Primary CGM result; study-specific window | D2 | 2.2 Were intervention providers aware of assigned intervention? | Yes | Randomized repeated intervention periods with at least one-week washout and objective CGM; result-specific n and marginal estimands were incompletely reported. | Low |
| de Hoogh 2025 | Primary CGM result; study-specific window | D2 | 2.3 Were there trial-context deviations beyond the intended protocol? | No | Randomized repeated intervention periods with at least one-week washout and objective CGM; result-specific n and marginal estimands were incompletely reported. | Low |
| de Hoogh 2025 | Primary CGM result; study-specific window | D2 | 2.4 Were any deviations unbalanced and likely to affect the result? | Not applicable | Randomized repeated intervention periods with at least one-week washout and objective CGM; result-specific n and marginal estimands were incompletely reported. | Low |
| de Hoogh 2025 | Primary CGM result; study-specific window | D2 | 2.5 Was an appropriate assigned-intervention analysis used? | Yes | Randomized repeated intervention periods with at least one-week washout and objective CGM; result-specific n and marginal estimands were incompletely reported. | Low |
| de Hoogh 2025 | Primary CGM result; study-specific window | D3 | 3.1 Were outcome data available for all or nearly all randomized participants/periods? | Probably no | Randomized repeated intervention periods with at least one-week washout and objective CGM; result-specific n and marginal estimands were incompletely reported. | Some concerns |
| de Hoogh 2025 | Primary CGM result; study-specific window | D3 | 3.2 If data were missing, was bias from missingness unlikely? | Probably no | Randomized repeated intervention periods with at least one-week washout and objective CGM; result-specific n and marginal estimands were incompletely reported. | Some concerns |
| de Hoogh 2025 | Primary CGM result; study-specific window | D4 | 4.1 Was the CGM/FGM measurement method appropriate? | Yes | Randomized repeated intervention periods with at least one-week washout and objective CGM; result-specific n and marginal estimands were incompletely reported. | Low |
| de Hoogh 2025 | Primary CGM result; study-specific window | D4 | 4.2 Could measurement or ascertainment differ between conditions? | No | Randomized repeated intervention periods with at least one-week washout and objective CGM; result-specific n and marginal estimands were incompletely reported. | Low |
| de Hoogh 2025 | Primary CGM result; study-specific window | D4 | 4.3 Could awareness of condition influence the recorded outcome? | No | Randomized repeated intervention periods with at least one-week washout and objective CGM; result-specific n and marginal estimands were incompletely reported. | Low |
| de Hoogh 2025 | Primary CGM result; study-specific window | D5 | 5.1 Was the reported analysis selected according to a prespecified plan? | No information | Randomized repeated intervention periods with at least one-week washout and objective CGM; result-specific n and marginal estimands were incompletely reported. | Some concerns |
| de Hoogh 2025 | Primary CGM result; study-specific window | D5 | 5.2 Was selection from multiple eligible outcome measurements unlikely? | No information | Randomized repeated intervention periods with at least one-week washout and objective CGM; result-specific n and marginal estimands were incompletely reported. | Some concerns |
| de Hoogh 2025 | Primary CGM result; study-specific window | D5 | 5.3 Was selection from multiple eligible analyses unlikely? | Probably no | Randomized repeated intervention periods with at least one-week washout and objective CGM; result-specific n and marginal estimands were incompletely reported. | Some concerns |
| Marcotte-Chénard 2024 | Primary CGM result; study-specific window | D1 | 1.1 Was the allocation sequence random? | No | Semi-randomized crossover: control was always first; exercise order was randomized; objective CGM; 14/15 complete. | High |
| Marcotte-Chénard 2024 | Primary CGM result; study-specific window | D1 | 1.2 Was the allocation sequence concealed until enrolment and assignment? | No | Semi-randomized crossover: control was always first; exercise order was randomized; objective CGM; 14/15 complete. | High |
| Marcotte-Chénard 2024 | Primary CGM result; study-specific window | D1 | 1.3 Did baseline or period-start differences suggest a problem with randomization? | Probably yes | Semi-randomized crossover: control was always first; exercise order was randomized; objective CGM; 14/15 complete. | High |
| Marcotte-Chénard 2024 | Primary CGM result; study-specific window | Period/carryover | S1 Was the crossover design suitable for this acute outcome? | Yes | Semi-randomized crossover: control was always first; exercise order was randomized; objective CGM; 14/15 complete. | High |
| Marcotte-Chénard 2024 | Primary CGM result; study-specific window | Period/carryover | S2 Was the washout sufficient to make important carryover unlikely? | Probably yes | Semi-randomized crossover: control was always first; exercise order was randomized; objective CGM; 14/15 complete. | High |
| Marcotte-Chénard 2024 | Primary CGM result; study-specific window | Period/carryover | S3 Were period/order effects controlled or shown to be negligible? | No | Semi-randomized crossover: control was always first; exercise order was randomized; objective CGM; 14/15 complete. | High |
| Marcotte-Chénard 2024 | Primary CGM result; study-specific window | D2 | 2.1 Were participants aware of their assigned intervention? | Yes | Semi-randomized crossover: control was always first; exercise order was randomized; objective CGM; 14/15 complete. | Low |
| Marcotte-Chénard 2024 | Primary CGM result; study-specific window | D2 | 2.2 Were intervention providers aware of assigned intervention? | Yes | Semi-randomized crossover: control was always first; exercise order was randomized; objective CGM; 14/15 complete. | Low |
| Marcotte-Chénard 2024 | Primary CGM result; study-specific window | D2 | 2.3 Were there trial-context deviations beyond the intended protocol? | No | Semi-randomized crossover: control was always first; exercise order was randomized; objective CGM; 14/15 complete. | Low |
| Marcotte-Chénard 2024 | Primary CGM result; study-specific window | D2 | 2.4 Were any deviations unbalanced and likely to affect the result? | Not applicable | Semi-randomized crossover: control was always first; exercise order was randomized; objective CGM; 14/15 complete. | Low |
| Marcotte-Chénard 2024 | Primary CGM result; study-specific window | D2 | 2.5 Was an appropriate assigned-intervention analysis used? | Yes | Semi-randomized crossover: control was always first; exercise order was randomized; objective CGM; 14/15 complete. | Low |
| Marcotte-Chénard 2024 | Primary CGM result; study-specific window | D3 | 3.1 Were outcome data available for all or nearly all randomized participants/periods? | Probably yes | Semi-randomized crossover: control was always first; exercise order was randomized; objective CGM; 14/15 complete. | Low |
| Marcotte-Chénard 2024 | Primary CGM result; study-specific window | D3 | 3.2 If data were missing, was bias from missingness unlikely? | Not applicable | Semi-randomized crossover: control was always first; exercise order was randomized; objective CGM; 14/15 complete. | Low |
| Marcotte-Chénard 2024 | Primary CGM result; study-specific window | D4 | 4.1 Was the CGM/FGM measurement method appropriate? | Yes | Semi-randomized crossover: control was always first; exercise order was randomized; objective CGM; 14/15 complete. | Low |
| Marcotte-Chénard 2024 | Primary CGM result; study-specific window | D4 | 4.2 Could measurement or ascertainment differ between conditions? | No | Semi-randomized crossover: control was always first; exercise order was randomized; objective CGM; 14/15 complete. | Low |
| Marcotte-Chénard 2024 | Primary CGM result; study-specific window | D4 | 4.3 Could awareness of condition influence the recorded outcome? | No | Semi-randomized crossover: control was always first; exercise order was randomized; objective CGM; 14/15 complete. | Low |
| Marcotte-Chénard 2024 | Primary CGM result; study-specific window | D5 | 5.1 Was the reported analysis selected according to a prespecified plan? | No information | Semi-randomized crossover: control was always first; exercise order was randomized; objective CGM; 14/15 complete. | Some concerns |
| Marcotte-Chénard 2024 | Primary CGM result; study-specific window | D5 | 5.2 Was selection from multiple eligible outcome measurements unlikely? | Probably no | Semi-randomized crossover: control was always first; exercise order was randomized; objective CGM; 14/15 complete. | Some concerns |
| Marcotte-Chénard 2024 | Primary CGM result; study-specific window | D5 | 5.3 Was selection from multiple eligible analyses unlikely? | No information | Semi-randomized crossover: control was always first; exercise order was randomized; objective CGM; 14/15 complete. | Some concerns |

Supporting information and the overall rationale are preserved in the locked audit workbook. Participant or provider non-blinding was not used alone to downgrade D2.

**Supplementary Figure S1. Model, correlation, core-definition, and alternative-arm sensitivity analyses for overall mean glucose**

Negative values favour activity. The paired-effect DL/normal model is a conventional sensitivity analysis calculated in R; REML-HKSJ is the primary inferential model.

**Supplementary Figure S2. Paired-effect REML-HKSJ forest plot for glucose coefficient of variation**

Study estimates are paired mean differences in percentage points.

**Supplementary Figure S3. Paired-effect REML-HKSJ forest plot for mean amplitude of glycaemic excursions**

Study estimates are paired mean differences in mmol/L.

**Supplementary Figure S4. Paired-effect REML-HKSJ forest plot for glucose standard deviation**

Study estimates are paired mean differences in mmol/L.

**Supplementary Figure S5. Paired-effect REML-HKSJ forest plot for time in range**

Study estimates are paired mean differences in percentage points; positive values favour activity. Because only two studies contributed to this analysis, the heterogeneity estimate and HKSJ interval should be interpreted cautiously.

**Supplementary Figure S6. Paired-effect REML-HKSJ forest plot for time above 10 mmol/L over 24 hours**

Only compatible >10 mmol/L, 24-hour, percentage-point results were combined. Terada minutes were converted by division by 14.4 with original values retained.

**Supplementary Figure S7. Paired-effect REML-HKSJ forest plot for time above 10 mmol/L over 22 hours**

The 22-hour stratum was analysed separately and was not relabelled as actual 24-hour exposure. Because only two studies contributed to this analysis, the heterogeneity estimate and HKSJ interval should be interpreted cautiously.

**Supplementary Figure S8. Leave-one-out REML-HKSJ analyses for overall mean glucose**

Each row refits the primary model after omitting one study. All leave-one-out confidence intervals excluded no effect.

**Supplementary Figure S9. Baujat diagnostics for overall mean glucose**

Dempsey 2017 was the principal contributor to heterogeneity and influence. The zoomed panel displays the remaining studies.

**Supplementary Figure S10. Influence diagnostics for overall mean glucose**

Four standard influence diagnostics—externally studentized residuals, Cook's distance, residual tau-squared after deletion, and DFBETAS for the pooled-effect intercept—identify Dempsey 2017 as influential; it was retained in the eligible primary model.

**Supplementary Figure S11. Contour-enhanced funnel plot for overall mean glucose**

The exploratory mixed-effects Egger-type test was non-significant (t[8]=-0.73; P=0.488). Exactly ten studies and high heterogeneity limit inference about missing-evidence bias.

**Supplementary Figure S12. Post hoc exploratory study-setting subgroup analysis for overall mean glucose**

Free-living or mixed-setting studies were internally consistent, whereas heterogeneity was concentrated among controlled laboratory studies. The formal interaction was not significant (F[1,8]=1.54; P=0.250), so setting was not established as an effect modifier.
