# Supplementary material for: Effects of short-bout accumulated physical activity and sedentary-break interventions on continuous glucose monitoring metrics in adults with type 2 diabetes: A systematic review and meta-analysis
Source: Front Endocrinol (Lausanne). 2026 Sep 11;17:1901259. doi: 10.3389/fendo.2026.1901259 (PMC13612268; doi:10.3389/fendo.2026.1901259)
Supplement: Supplementary file 2 [file Supplementaryfile2.docx]

**Supplementary File: Complete Search Strategies**

Search chronology: the original eight-database strategies were executed on June 2–4, 2026 and combined population, intervention, outcome, and study-design concepts. Before finalizing the review, the strategies were broadened by removing the outcome concept and expanding coverage to eleven bibliographic and registry sources. The revised searches shown below were rerun from database inception on July 29, 2026. They used population plus intervention concepts, with a study-design concept in bibliographic databases where appropriate. CENTRAL and trial registries were searched without an additional randomized-trial filter. The original submitted strategies and their database-specific execution dates are reproduced in Supplementary Appendix A.

# Search-source summary

| **Source** | **Interface** | **Records** | **Limits/export evidence** |
| --- | --- | --- | --- |
| MEDLINE via PubMed | PubMed | 496 | No language or publication-status limits; animal-only records excluded; PubMed records and execution metadata |
| Embase | Embase.com | 986 | No language or publication-status limits; animal-only records excluded; Embase full-record text export |
| Scopus | Scopus | 1160 | No language, publication-status, document-type, or subject-area limits; Scopus full-record text export |
| Web of Science Core Collection | Web of Science | 645 | All years; no language, publication-status, or document-type limits; Web of Science tagged plain text |
| Cochrane Central Register of Controlled Trials (CENTRAL) | Cochrane Library | 694 | CENTRAL Trials only; no language, date, or publication-status limits; Cochrane Library text export |
| CINAHL Ultimate | EBSCOhost | 193 | No filters applied; CSV in four batches |
| APA PsycInfo | EBSCOhost | 33 | No filters applied; CSV |
| SPORTDiscus with Full Text | EBSCOhost | 127 | No language, date, peer-review, or full-text filter; CSV in three batches |
| China National Knowledge Infrastructure (CNKI) | CNKI Professional Search | 9 | No language, date, funding, or publication-status limits; CNKI plain-text full records |
| ClinicalTrials.gov | ClinicalTrials.gov API v2 | 101 | No recruitment-status or study-type filter at search stage; JSON and CSV |
| WHO International Clinical Trials Registry Platform (ICTRP) | WHO ICTRP Search Portal | 148 | No recruitment-status, phase, results-availability, or language limits; XML; 148 unique trial IDs |

Note: the Cochrane Library displayed 695 total results (694 CENTRAL trial records and one Cochrane Review); 694 was used for PRISMA. WHO ICTRP displayed 150 registry records representing 148 unique trials; 148 was used for PRISMA. Across all sources, the locked total was 4,592 records.

# 1. MEDLINE via PubMed

**Interface:** PubMed **| Records:** 496 **| Limits:** No language or publication-status limits; animal-only records excluded **| Export:** PubMed records and execution metadata

**Exact executed strategy**

(("Diabetes Mellitus, Type 2"[Mesh] OR "type 2 diabetes"[tiab] OR "type II diabetes"[tiab] OR "type 2 diabetic*"[tiab] OR "type II diabetic*"[tiab] OR T2D[tiab] OR T2DM[tiab] OR NIDDM[tiab] OR "noninsulin dependent diabetes"[tiab] OR "non-insulin-dependent diabetes"[tiab])) AND (("exercise snack*"[tiab] OR "activity snack*"[tiab] OR "physical activity snack*"[tiab] OR "activity break*"[tiab] OR "movement break*"[tiab] OR "sedentary break*"[tiab] OR "sitting break*"[tiab] OR (interrupt*[tiab] AND sitting[tiab]) OR (break*[tiab] AND sitting[tiab]) OR "prolonged sitting"[tiab] OR "postprandial walk*"[tiab] OR "postmeal walk*"[tiab] OR "post-meal walk*"[tiab] OR "walking after meal*"[tiab] OR "postprandial exercise"[tiab] OR "post-meal exercise"[tiab] OR "exercise timing"[tiab] OR "accumulated exercise"[tiab] OR "accumulated physical activity"[tiab] OR "short bout*"[tiab] OR "brief bout*"[tiab] OR "intermittent activit*"[tiab] OR "intermittent exercise"[tiab] OR "simple resistance activit*"[tiab] OR "light-intensity walk*"[tiab] OR "activities of daily living"[tiab] OR "high-intensity interval*"[tiab] OR "low-volume interval*"[tiab] OR "sprint interval*"[tiab] OR "reduced-exertion high-intensity"[tiab] OR REHIT[tiab] OR HIIT[tiab] OR "interval exercise"[tiab] OR "interval training"[tiab] OR lunchtime[tiab])) AND ((randomized controlled trial[pt] OR controlled clinical trial[pt] OR randomized[tiab] OR randomised[tiab] OR randomly[tiab] OR trial[tiab] OR crossover[tiab] OR cross-over[tiab] OR "cross over"[tiab] OR controlled[tiab] OR intervention*[tiab] OR "repeated measures"[tiab]) NOT (animals[mh] NOT humans[mh]))

# 2. Embase

**Interface:** Embase.com **| Records:** 986 **| Limits:** No language or publication-status limits; animal-only records excluded **| Export:** Embase full-record text export

**Exact executed strategy**

#1
'type 2 diabetes mellitus'/exp OR ('type 2 diabetes' OR 'type ii diabetes' OR 'type 2 diabetic*' OR 'type ii diabetic*' OR t2d OR t2dm OR niddm OR 'non insulin dependent diabetes'):ti,ab,kw

#2
('exercise snack*' OR 'activity snack*' OR 'physical activity snack*' OR 'activity break*' OR 'movement break*' OR 'sedentary break*' OR 'sitting break*' OR (interrupt* NEAR/3 sitting) OR (break* NEAR/3 sitting) OR 'prolonged sitting' OR 'postprandial walk*' OR 'postmeal walk*' OR 'post meal walk*' OR (walking NEAR/3 meal*) OR 'postprandial exercise' OR 'post meal exercise' OR 'exercise timing' OR 'accumulated exercise' OR 'accumulated physical activity' OR 'short bout*' OR 'brief bout*' OR 'intermittent activit*' OR 'intermittent exercise' OR 'simple resistance activit*' OR 'light intensity walk*' OR 'activities of daily living' OR 'high intensity interval*' OR 'low volume interval*' OR 'sprint interval*' OR 'reduced exertion high intensity' OR rehit OR hiit OR 'interval exercise' OR 'interval training' OR lunchtime):ti,ab,kw

#3
'randomized controlled trial'/exp OR 'controlled clinical trial'/exp OR 'crossover procedure'/exp OR (random* OR trial OR crossover* OR 'cross over' OR controlled OR intervention* OR 'repeated measure*'):ti,ab,kw

#4
#1 AND #2 AND #3

#5
#4 NOT ('animal'/exp NOT 'human'/exp)

# 3. Scopus

**Interface:** Scopus **| Records:** 1160 **| Limits:** No language, publication-status, document-type, or subject-area limits **| Export:** Scopus full-record text export

**Exact executed strategy**

TITLE-ABS-KEY("type 2 diabetes" OR "type II diabetes" OR "type 2 diabetic*" OR "type II diabetic*" OR T2D OR T2DM OR NIDDM OR "non insulin dependent diabetes")
AND
TITLE-ABS-KEY("exercise snack*" OR "activity snack*" OR "physical activity snack*" OR "activity break*" OR "movement break*" OR "sedentary break*" OR "sitting break*" OR (interrupt* W/3 sitting) OR (break* W/3 sitting) OR "prolonged sitting" OR "postprandial walk*" OR "postmeal walk*" OR "post meal walk*" OR (walking W/3 meal*) OR "postprandial exercise" OR "post meal exercise" OR "exercise timing" OR "accumulated exercise" OR "accumulated physical activity" OR "short bout*" OR "brief bout*" OR "intermittent activit*" OR "intermittent exercise" OR "simple resistance activit*" OR "light intensity walk*" OR "activities of daily living" OR "high intensity interval*" OR "low volume interval*" OR "sprint interval*" OR "reduced exertion high intensity" OR REHIT OR HIIT OR "interval exercise" OR "interval training" OR lunchtime)
AND
TITLE-ABS-KEY(random* OR trial OR crossover* OR "cross over" OR controlled OR intervention* OR "repeated measure*")

# 4. Web of Science Core Collection

**Interface:** Web of Science **| Records:** 645 **| Limits:** All years; no language, publication-status, or document-type limits **| Export:** Web of Science tagged plain text

**Exact executed strategy**

TS=("type 2 diabetes" OR "type II diabetes" OR "type 2 diabetic*" OR "type II diabetic*" OR T2D OR T2DM OR NIDDM OR "non insulin dependent diabetes")
AND
TS=("exercise snack*" OR "activity snack*" OR "physical activity snack*" OR "activity break*" OR "movement break*" OR "sedentary break*" OR "sitting break*" OR (interrupt* NEAR/3 sitting) OR (break* NEAR/3 sitting) OR "prolonged sitting" OR "postprandial walk*" OR "postmeal walk*" OR "post meal walk*" OR (walking NEAR/3 meal*) OR "postprandial exercise" OR "post meal exercise" OR "exercise timing" OR "accumulated exercise" OR "accumulated physical activity" OR "short bout*" OR "brief bout*" OR "intermittent activit*" OR "intermittent exercise" OR "simple resistance activit*" OR "light intensity walk*" OR "activities of daily living" OR "high intensity interval*" OR "low volume interval*" OR "sprint interval*" OR "reduced exertion high intensity" OR REHIT OR HIIT OR "interval exercise" OR "interval training" OR lunchtime)
AND
TS=(random* OR trial OR crossover* OR "cross over" OR controlled OR intervention* OR "repeated measure*")

# 5. Cochrane Central Register of Controlled Trials (CENTRAL)

**Interface:** Cochrane Library **| Records:** 694 **| Limits:** CENTRAL Trials only; no language, date, or publication-status limits **| Export:** Cochrane Library text export

**Exact executed strategy**

#1 MeSH descriptor: [Diabetes Mellitus, Type 2] explode all trees
#2 ("type 2 diabetes" OR "type II diabetes" OR (type NEXT 2 NEXT diabetic*) OR (type NEXT II NEXT diabetic*) OR T2D OR T2DM OR NIDDM OR "non insulin dependent diabetes"):ti,ab,kw
#3 #1 OR #2
#4 ((exercise NEXT snack*) OR (activity NEXT snack*) OR (physical NEXT activity NEXT snack*) OR (activity NEXT break*) OR (movement NEXT break*) OR (sedentary NEXT break*) OR (sitting NEXT break*) OR (interrupt* NEAR/3 sitting) OR (break* NEAR/3 sitting) OR "prolonged sitting" OR (postprandial NEXT walk*) OR (postmeal NEXT walk*) OR (post NEXT meal NEXT walk*) OR (walking NEAR/3 meal*) OR "postprandial exercise" OR "post meal exercise" OR "exercise timing" OR "accumulated exercise" OR "accumulated physical activity" OR (short NEXT bout*) OR (brief NEXT bout*) OR (intermittent NEXT activit*) OR "intermittent exercise" OR (simple NEXT resistance NEXT activit*) OR (light NEXT intensity NEXT walk*) OR "activities of daily living" OR (high NEXT intensity NEXT interval*) OR (low NEXT volume NEXT interval*) OR (sprint NEXT interval*) OR "reduced exertion high intensity" OR REHIT OR HIIT OR "interval exercise" OR "interval training" OR lunchtime):ti,ab,kw
#5 #3 AND #4

# 6. CINAHL Ultimate

**Interface:** EBSCOhost **| Records:** 193 **| Limits:** No filters applied **| Export:** CSV in four batches

**Exact executed strategy**

((MH "Diabetes Mellitus, Type 2+") OR TI ("type 2 diabetes" OR "type II diabetes" OR (type W1 2 W1 diabetic*) OR (type W1 II W1 diabetic*) OR T2D OR T2DM OR NIDDM OR "non insulin dependent diabetes") OR AB ("type 2 diabetes" OR "type II diabetes" OR (type W1 2 W1 diabetic*) OR (type W1 II W1 diabetic*) OR T2D OR T2DM OR NIDDM OR "non insulin dependent diabetes")) AND (TI ((exercise W1 snack*) OR (activity W1 snack*) OR (physical W1 activity W1 snack*) OR (activity W1 break*) OR (movement W1 break*) OR (sedentary W1 break*) OR (sitting W1 break*) OR (interrupt* N3 sitting) OR (break* N3 sitting) OR "prolonged sitting" OR (postprandial N3 walk*) OR (postmeal N3 walk*) OR (post W1 meal N3 walk*) OR (walking N3 meal*) OR "postprandial exercise" OR "post meal exercise" OR "exercise timing" OR "accumulated exercise" OR "accumulated physical activity" OR (short W1 bout*) OR (brief W1 bout*) OR (intermittent W1 activit*) OR "intermittent exercise" OR (simple W1 resistance W1 activit*) OR (light W1 intensity N3 walk*) OR "activities of daily living" OR (high W1 intensity W1 interval*) OR (low W1 volume W1 interval*) OR (sprint W1 interval*) OR "reduced exertion high intensity" OR REHIT OR HIIT OR "interval exercise" OR "interval training" OR lunchtime) OR AB ((exercise W1 snack*) OR (activity W1 snack*) OR (physical W1 activity W1 snack*) OR (activity W1 break*) OR (movement W1 break*) OR (sedentary W1 break*) OR (sitting W1 break*) OR (interrupt* N3 sitting) OR (break* N3 sitting) OR "prolonged sitting" OR (postprandial N3 walk*) OR (postmeal N3 walk*) OR (post W1 meal N3 walk*) OR (walking N3 meal*) OR "postprandial exercise" OR "post meal exercise" OR "exercise timing" OR "accumulated exercise" OR "accumulated physical activity" OR (short W1 bout*) OR (brief W1 bout*) OR (intermittent W1 activit*) OR "intermittent exercise" OR (simple W1 resistance W1 activit*) OR (light W1 intensity N3 walk*) OR "activities of daily living" OR (high W1 intensity W1 interval*) OR (low W1 volume W1 interval*) OR (sprint W1 interval*) OR "reduced exertion high intensity" OR REHIT OR HIIT OR "interval exercise" OR "interval training" OR lunchtime)) AND (TI (random* OR trial OR crossover* OR "cross over" OR controlled OR intervention* OR (repeated W1 measure*)) OR AB (random* OR trial OR crossover* OR "cross over" OR controlled OR intervention* OR (repeated W1 measure*)))

# 7. APA PsycInfo

**Interface:** EBSCOhost **| Records:** 33 **| Limits:** No filters applied **| Export:** CSV

**Exact executed strategy**

((DE "Type 2 Diabetes" OR DE "Diabetes Mellitus" OR TI ("type 2 diabetes" OR "type II diabetes" OR (type W1 2 W1 diabetic*) OR (type W1 II W1 diabetic*) OR T2D OR T2DM OR NIDDM OR "non insulin dependent diabetes") OR AB ("type 2 diabetes" OR "type II diabetes" OR (type W1 2 W1 diabetic*) OR (type W1 II W1 diabetic*) OR T2D OR T2DM OR NIDDM OR "non insulin dependent diabetes")) AND (TI ((exercise W1 snack*) OR (activity W1 snack*) OR (physical W1 activity W1 snack*) OR (activity W1 break*) OR (movement W1 break*) OR (sedentary W1 break*) OR (sitting W1 break*) OR (interrupt* N3 sitting) OR (break* N3 sitting) OR "prolonged sitting" OR (postprandial N3 walk*) OR (postmeal N3 walk*) OR (post W1 meal N3 walk*) OR (walking N3 meal*) OR "postprandial exercise" OR "post meal exercise" OR "exercise timing" OR "accumulated exercise" OR "accumulated physical activity" OR (short W1 bout*) OR (brief W1 bout*) OR (intermittent W1 activit*) OR "intermittent exercise" OR (simple W1 resistance W1 activit*) OR (light W1 intensity N3 walk*) OR "activities of daily living" OR (high W1 intensity W1 interval*) OR (low W1 volume W1 interval*) OR (sprint W1 interval*) OR "reduced exertion high intensity" OR REHIT OR HIIT OR "interval exercise" OR "interval training" OR lunchtime) OR AB ((exercise W1 snack*) OR (activity W1 snack*) OR (physical W1 activity W1 snack*) OR (activity W1 break*) OR (movement W1 break*) OR (sedentary W1 break*) OR (sitting W1 break*) OR (interrupt* N3 sitting) OR (break* N3 sitting) OR "prolonged sitting" OR (postprandial N3 walk*) OR (postmeal N3 walk*) OR (post W1 meal N3 walk*) OR (walking N3 meal*) OR "postprandial exercise" OR "post meal exercise" OR "exercise timing" OR "accumulated exercise" OR "accumulated physical activity" OR (short W1 bout*) OR (brief W1 bout*) OR (intermittent W1 activit*) OR "intermittent exercise" OR (simple W1 resistance W1 activit*) OR (light W1 intensity N3 walk*) OR "activities of daily living" OR (high W1 intensity W1 interval*) OR (low W1 volume W1 interval*) OR (sprint W1 interval*) OR "reduced exertion high intensity" OR REHIT OR HIIT OR "interval exercise" OR "interval training" OR lunchtime)) AND (TI (random* OR trial OR crossover* OR "cross over" OR controlled OR intervention* OR (repeated W1 measure*)) OR AB (random* OR trial OR crossover* OR "cross over" OR controlled OR intervention* OR (repeated W1 measure*))))

# 8. SPORTDiscus with Full Text

**Interface:** EBSCOhost **| Records:** 127 **| Limits:** No language, date, peer-review, or full-text filter **| Export:** CSV in three batches

**Exact executed strategy**

((SU ("type 2 diabetes" OR "type II diabetes" OR "diabetes mellitus, type 2" OR T2D OR T2DM OR NIDDM OR "non insulin dependent diabetes") OR TI ("type 2 diabetes" OR "type II diabetes" OR (type W1 2 W1 diabetic*) OR (type W1 II W1 diabetic*) OR T2D OR T2DM OR NIDDM OR "non insulin dependent diabetes") OR AB ("type 2 diabetes" OR "type II diabetes" OR (type W1 2 W1 diabetic*) OR (type W1 II W1 diabetic*) OR T2D OR T2DM OR NIDDM OR "non insulin dependent diabetes")) AND (TI ((exercise W1 snack*) OR (activity W1 snack*) OR (physical W1 activity W1 snack*) OR (activity W1 break*) OR (movement W1 break*) OR (sedentary W1 break*) OR (sitting W1 break*) OR (interrupt* N3 sitting) OR (break* N3 sitting) OR "prolonged sitting" OR (postprandial N3 walk*) OR (postmeal N3 walk*) OR (post W1 meal N3 walk*) OR (walking N3 meal*) OR "postprandial exercise" OR "post meal exercise" OR "exercise timing" OR "accumulated exercise" OR "accumulated physical activity" OR (short W1 bout*) OR (brief W1 bout*) OR (intermittent W1 activit*) OR "intermittent exercise" OR (simple W1 resistance W1 activit*) OR (light W1 intensity N3 walk*) OR "activities of daily living" OR (high W1 intensity W1 interval*) OR (low W1 volume W1 interval*) OR (sprint W1 interval*) OR "reduced exertion high intensity" OR REHIT OR HIIT OR "interval exercise" OR "interval training" OR lunchtime) OR AB ((exercise W1 snack*) OR (activity W1 snack*) OR (physical W1 activity W1 snack*) OR (activity W1 break*) OR (movement W1 break*) OR (sedentary W1 break*) OR (sitting W1 break*) OR (interrupt* N3 sitting) OR (break* N3 sitting) OR "prolonged sitting" OR (postprandial N3 walk*) OR (postmeal N3 walk*) OR (post W1 meal N3 walk*) OR (walking N3 meal*) OR "postprandial exercise" OR "post meal exercise" OR "exercise timing" OR "accumulated exercise" OR "accumulated physical activity" OR (short W1 bout*) OR (brief W1 bout*) OR (intermittent W1 activit*) OR "intermittent exercise" OR (simple W1 resistance W1 activit*) OR (light W1 intensity N3 walk*) OR "activities of daily living" OR (high W1 intensity W1 interval*) OR (low W1 volume W1 interval*) OR (sprint W1 interval*) OR "reduced exertion high intensity" OR REHIT OR HIIT OR "interval exercise" OR "interval training" OR lunchtime)) AND (TI (random* OR trial OR crossover* OR "cross over" OR controlled OR intervention* OR (repeated W1 measure*)) OR AB (random* OR trial OR crossover* OR "cross over" OR controlled OR intervention* OR (repeated W1 measure*))))

# 9. China National Knowledge Infrastructure (CNKI)

**Interface:** CNKI Professional Search **| Records:** 9 **| Limits:** No language, date, funding, or publication-status limits **| Export:** CNKI plain-text full records

**Exact executed strategy**

SU=('2型糖尿病'+'Ⅱ型糖尿病'+'II型糖尿病'+'T2D'+'T2DM'+'非胰岛素依赖型糖尿病') AND SU=('运动零食'+'运动点心'+'活动零食'+'短时运动'+'短时身体活动'+'短时体力活动'+'累积运动'+'累积身体活动'+'累积体力活动'+'分段运动'+'活动间歇'+'运动间歇'+'久坐打断'+'久坐中断'+'久坐间断'+'久坐休息'+'打断久坐'+'餐后步行'+'餐后运动'+'饭后步行'+'饭后运动'+'简单抗阻活动'+'轻强度步行'+'日常生活活动'+'高强度间歇训练'+'低容量间歇训练'+'冲刺间歇训练'+'HIIT'+'REHIT'+'间歇运动'+'exercise snack'+'activity snack'+'sedentary break'+'postprandial walking'+'high intensity interval training') AND SU=('随机'+'随机对照'+'交叉'+'自身对照'+'对照试验'+'临床试验'+'干预研究'+'重复测量'+'random'+'randomized'+'randomised'+'crossover'+'trial')

# 10. ClinicalTrials.gov

**Interface:** ClinicalTrials.gov API v2 **| Records:** 101 **| Limits:** No recruitment-status or study-type filter at search stage **| Export:** JSON and CSV

**Exact executed strategy**

Condition/disease：
("type 2 diabetes" OR T2D OR T2DM)

Intervention/treatment：
("exercise snack" OR "activity snack" OR "physical activity snack" OR "activity break" OR "movement break" OR "sedentary break" OR "sitting break" OR "interrupting sitting" OR "breaks in sitting" OR "prolonged sitting" OR "postprandial walking" OR "post-meal walking" OR "walking after meals" OR "postprandial exercise" OR "exercise timing" OR "accumulated exercise" OR "accumulated physical activity" OR "short bout" OR "brief bout" OR "intermittent exercise" OR "simple resistance activities" OR "light-intensity walking" OR "activities of daily living" OR "high-intensity interval" OR "low-volume interval" OR "sprint interval" OR "reduced-exertion high-intensity" OR REHIT OR HIIT OR "interval exercise" OR "interval training")

# 11. WHO International Clinical Trials Registry Platform (ICTRP)

**Interface:** WHO ICTRP Search Portal **| Records:** 148 **| Limits:** No recruitment-status, phase, results-availability, or language limits **| Export:** XML; 148 unique trial IDs

**Exact executed strategy**

("type 2 diabetes" OR "type II diabetes" OR "type 2 diabetic" OR "type II diabetic" OR T2D OR T2DM OR NIDDM OR "non insulin dependent diabetes") AND ("exercise snack" OR "exercise snacks" OR "activity snack" OR "activity snacks" OR "physical activity snack" OR "activity break" OR "movement break" OR "sedentary break" OR "sitting break" OR "interrupt sitting" OR "break sitting" OR "prolonged sitting" OR "postprandial walking" OR "postmeal walking" OR "post meal walking" OR "walking after meal" OR "postprandial exercise" OR "post meal exercise" OR "exercise timing" OR "accumulated exercise" OR "accumulated physical activity" OR "short bout exercise" OR "brief bout exercise" OR "intermittent activity" OR "intermittent exercise" OR "simple resistance activities" OR "light intensity walking" OR "activities of daily living" OR "high intensity interval training" OR "low volume interval training" OR "sprint interval training" OR "reduced exertion high intensity" OR REHIT OR HIIT OR "interval exercise" OR "interval training" OR lunchtime)
